# Multiomics and machine learning-based analysis of pancancer pseudouridine modifications

**DOI:** 10.1007/s12672-024-01093-y

**Published:** 2024-08-20

**Authors:** Jiheng Zhang, Lei Xu, Xiuwei Yan, Jiahe Hu, Xin Gao, Hongtao Zhao, Mo Geng, Nan Wang, Shaoshan Hu

**Affiliations:** 1 Cancer Center, Department of Neurosurgery, Zhejiang Provincial People’s Hospital (Affiliated People’s Hospital), Hangzhou Medical College, Hangzhou, Zhejiang China; 2 Department of Neurosurgery, Peking Union Medical College Hospital, Chinese Academy of Medical Sciences and Peking Union Medical College, Beijing, China; 3 Department of Neurosurgery, Beijing Tiantan Hospital, Capital Medical University, Beijing, China

**Keywords:** Pseudouridine modification, Pancancer, Tumor microenvironment, Machine learning, Antitumor therapy

## Abstract

Pseudouridine widely affects the stability and function of RNA. However, our knowledge of pseudouridine properties in tumors is incomplete. We systematically analyzed pseudouridine synthases (PUSs) expression, genomic aberrations, and prognostic features in 10907 samples from 33 tumors. We found that the pseudouridine-associated pathway was abnormal in tumors and affected patient prognosis. Dysregulation of the PUSs expression pattern may arise from copy number variation (CNV) mutations and aberrant DNA methylation. Functional enrichment analyses determined that the PUSs expression was closely associated with the MYC, E2F, and MTORC1 signaling pathways. In addition, PUSs are involved in the remodeling of the tumor microenvironment (TME) in solid tumors, such as kidney and lung cancers. Particularly in lung cancer, increased expression of PUSs is accompanied by increased immune checkpoint expression and Treg infiltration. The best signature model based on more than 112 machine learning combinations had good prognostic ability in ACC, DLBC, GBM, KICH, MESO, THYM, TGCT, and PRAD tumors, and is expected to guide immunotherapy for 19 tumor types. The model was also effective in identifying patients with tumors amenable to etoposide, camptothecin, cisplatin, or bexarotene treatment. In conclusion, our work highlights the dysregulated features of PUSs and their role in the TME and patient prognosis, providing an initial molecular basis for future exploration of pseudouridine. Studies targeting pseudouridine are expected to lead to the development of potential diagnostic strategies and the evaluation and improvement of antitumor therapies.

**Supplementary Information:**

The online version contains supplementary material available at 10.1007/s12672-024-01093-y.

## Introduction

Cancer constitutes a serious threat to human health, and tens of millions of people die of cancer every year [1]. Despite the rapid development of chemotherapy and immunotherapy treatments in recent years, which have greatly prolonged the survival of patients with tumors, the overall prognosis of these patients still falls short of expectations [2, 3]. Tremendous knowledge has been accumulated regarding genomics and molecular mechanisms, and it has been found that tumor development and progression are associated with the dysregulation of genes [4]. Therefore, there is an urgent need for in-depth research into the causes of dysregulated genes and transcriptional analyses targeting specific molecules to improve cancer diagnoses and individualized treatment options.

Notably, RNA alterations can be catalyzed by several strictly substrate-specific enzymes, leading to posttranscriptional modifications. Dysregulation of these enzymes can lead to several serious diseases, including cancer [5]. Pseudouridine, one of the most abundant RNA modifications in human cells, is mediated by pseudouridine synthases (PUSs), which alter the chemical structure of uracil nucleotides. This process maintains RNA stability and function, thereby affecting downstream signaling [6, 7]. Dysregulated PUSs promote tumor progression and impact patient prognoses. Significantly increased expression of the PUSs PUS1 and PUS7 was observed in kidney clear cell carcinoma (KIRC) [8]. In addition, patients with hepatocellular carcinoma who demonstrate upregulated PUS1 and PUS7 expression have decreased overall survival (OS) [9]. In colorectal cancer (CRC) patients, depletion of the synthetase DKC1 leads to increased attenuation of the mRNA expression of several ribosomal proteins, thereby mediating tumor cell growth [10]. Similar to RNA methylation, such as the addition of 5-methylcytosine and N6-methyladenosine, pseudouridine can also occur in mRNAs, rRNAs, and tRNAs; however, the difference is that the pseudouridine synthesis process is irreversible [11, 12]. Therefore, aberrant pseudouridine modifications appear to have more serious consequences. However, most studies on pseudouridine are based on individual genes or single tumors. Pancancer and multiomics systematic analyses of PUSs that assess their impact on multiple cancers and their clinical applications are lacking.

Compelling evidence suggests that pseudouridine modifications are critical for therapeutic resistance. Pyrazofuran, a small molecule inhibitor of DKC1, inhibits CRC growth [10]. Given the complex regulation of pseudouridine in cancer progression and its important impact on anticancer therapy, we performed comprehensive genomic and functional analyses of PUSs across cancer types and calculated the impacts of their roles in the tumor immune microenvironment. The results revealed that PUSs tend to drive oncogenic effects and are involved in immune cell infiltration and immune function. Subsequently, we screened and constructed a PUSs-related model using 112 machine learning methods, which was able to stably predict the prognosis of a wide range of tumors and efficiently identify patients who would benefit from chemotherapy and immunotherapy.

## Materials and methods

### Sample data sources

By reviewing several high-quality articles on pseudouridine, we identified eight genes, namely PUS1, PUS3, PUS7, PUS10, RPUSD4, DKC1, TRUB1, and TRUB2, as targets for our study [13–17].

The convenient UCSC Xena website (https://xena.ucsc.edu/) integrates numerous publicly publishable sequencing data, including information on 33 tumors and normal samples from the TCGA database and expression profiles of normal samples from the GTEx database [18]. On this basis, we obtained mRNA expression profiles of tumor samples and matched normal samples, and analyzed them after excluding batch effects to ensure data uniformity and compatibility. We collated the number of samples for each cancer type and the corresponding normal samples from GTEx and have displayed them in Supplementary Table 1. After excluding samples without OS information, we retained 10105 pancancer samples for subsequent prognostic analyses (including survival analyses and signature model construction). We downloaded mutation data for all tumor samples using the R package ‘TCGAbiolinks’ and analyzed and revisualized them using the ‘maftools’ package. Furthermore, we calculated the tumor mutation burden (TMB) and microsatellite instability (MSI) data of these samples as markers for determining treatment response. CNV data for pancancer samples were collected and collated from the TCGA database and the UCSC website, and estimates were generated using the GISTIC2 method. We used Spearman correlation analysis to determine the relationship between CNV variation and the mRNA expression of PUSs. The GSCA data analysis platform processes and summarizes tumor DNA methylation data (http://bioinfo.life.hust.edu.cn/GSCA) [19]. Therefore, we explored the strength of the association between the activities of PUSs and their methylation status.

### Functional enrichment analysis

We obtained signature gene sets, including pseudouridine modification-related pathways and ‘hallmark gene sets’ from MSigDB (https://www.gsea-msigdb.org/gsea/msigdb), and used the single-sample genomic enrichment analysis (ssGSEA) algorithm to characterize the degree of activation of these pathways in all types of tumors [20]. Finally, Spearman correlation analysis was used to quantify the extent to which different PUSs were associated with all pathways.

### Immunoinformatic analysis

TIMER2.0 was used to analyze the abundance of six immune cell infiltration levels in different cancer types using the RNA-seq expression profiling data system [21]. We analyzed the TIMER2.0 data and calculated the correlation between the eight PUSs and the six immune infiltration scores using Spearman correlation analysis. Jia et al. refined the landscape of immune cell infiltration in the tumor microenvironment (TME) and summarized the characteristic genes for each cell type [22]. We next quantified and visualized immune cell infiltration in lung cancer based on the ssGSEA algorithm and GSCA. Different strategies have been used to infer the impact of PUSs on the TME. Finally, we explored the expression patterns of the immune checkpoints (ICPs) PD-1, PD-L1, and CTLA4 under the influence of PUSs to statistically test the functions of immune cells.

### Construction of pseudouridine signature scores

To comprehensively better describe the effects of pseudouridine on tumors, we constructed a pseudouridine signature score. To construct the best characterization model, the underlying factors should be small enough to have the potential for practical clinical application yet general and representative enough to integrate important information. This is a great challenge when faced with complex data from multiple tumor types. First, we screened all genes associated with the expression of the eight PUSs in 33 tumors based on Pearson correlation analysis (screening criteria | cor |> 0.5 and p < 0.05). Immediately thereafter, we identified a total of 21 genes across all tumor types that maintained consistent correlations with expression of the corresponding PUSs. We then followed the methodology of a high-quality article [23]. By integrating 12 machine learning algorithms, including random forest (RF), elasticity network (Enet), LASSO, ridge, naïveBayes, Stepglm, glmBoost, plsRglm, XGBoost, LDA, generalized boosted regression model (GBM) and survival support vector machine (SVM), we generated a consensus model (Supplementary method). Using the leave-one-out cross-validation (LOOCV) framework, we used a total of 112 algorithm combinations to fit the predictive model. To identify the best model, we constructed a test datasetfrom each tumor and then applied it to other tumors for training. After repeating the calculation 33 times, the predictive power of each model was assessed using the average C-index. The highest C-index of the feature model was ultimately constructed using the tumor ACC as a template. Five genes, including CEBPZ, TIAL1, COX15, NKAPD1, and DNAJC2 were used to construct the characterisation model with glmBoost + Enet [alpha = 0.2]. The pseudouridine signature scores = 1.140369 × CEBPZ^**exp**^ + 1.615863 × TIAL1^**exp**^ + (− 2.161033)** × **COX15^**exp**^ + 0.4450443 × NKAPD1^**exp**^ + 0.3082516 × DNAJC2^**exp**^. We normalized the final signature scores and projected them to be between 0 and 1. Patients with scores less than 0.5 were labeled low signature scorers for subsequent stratified training.

### Chemotherapy drug sensitivity

The ‘pRRophetic’ package has been very useful for identifying potential associations between drug sensitivity and the genome. The method builds a model with the help of known gene expression matrices and drug sensitivities from over 500 cell lines for the training set, and then fits a ridge regression to obtain the predicted values by normalizing the gene expression of the training and test sets, using each gene as a predictor variable and the IC50 of the drug as the outcome variable [24, 25]. This method has been used in many high-quality articles and is one of the reliable means of prediction [26, 27]. Using this package, we predicted the extent of differences in the IC50 values of chemotherapeutic drugs in high pseudouridine signature cohorts and low pseudouridine signature cohorts of different tumor samples by performing ridge regression analyses and incorporating the expression profiles of each tumor.

### Immunotherapy responses

The TIDE (http://tid.dfci.harvard.edu) decision tool integrates multiple published pretreatment expression profiles of tumors [28]. The algorithm is able to predict a patient's response to immune checkpoint blockade (ICB) therapies based on our collated tumor expression profiles.

### Statistical analysis

All the statistical analyses and visualizations were performed in R (version 4.1.2). High and low groupings were made based on the median gene expression of the respective PUSs for each tumor. In addition, the high- and low-pseudouridine signature score groups were cut off at 0.5. Kaplan‒Meier (K‒M) analysis and log-rank tests were used to compare differences in survival between groups. The Wilcoxon test was used to compare differences in gene expression within subgroups. Independent prognostic values were assessed using univariate Cox regression analyses. Fisher's exact test was used to determine differences in response to ICB therapy between subgroups with different pseudouridine signatures.

## Results

### Abnormal pseudouridine-related pathways

**Fig. 1:**
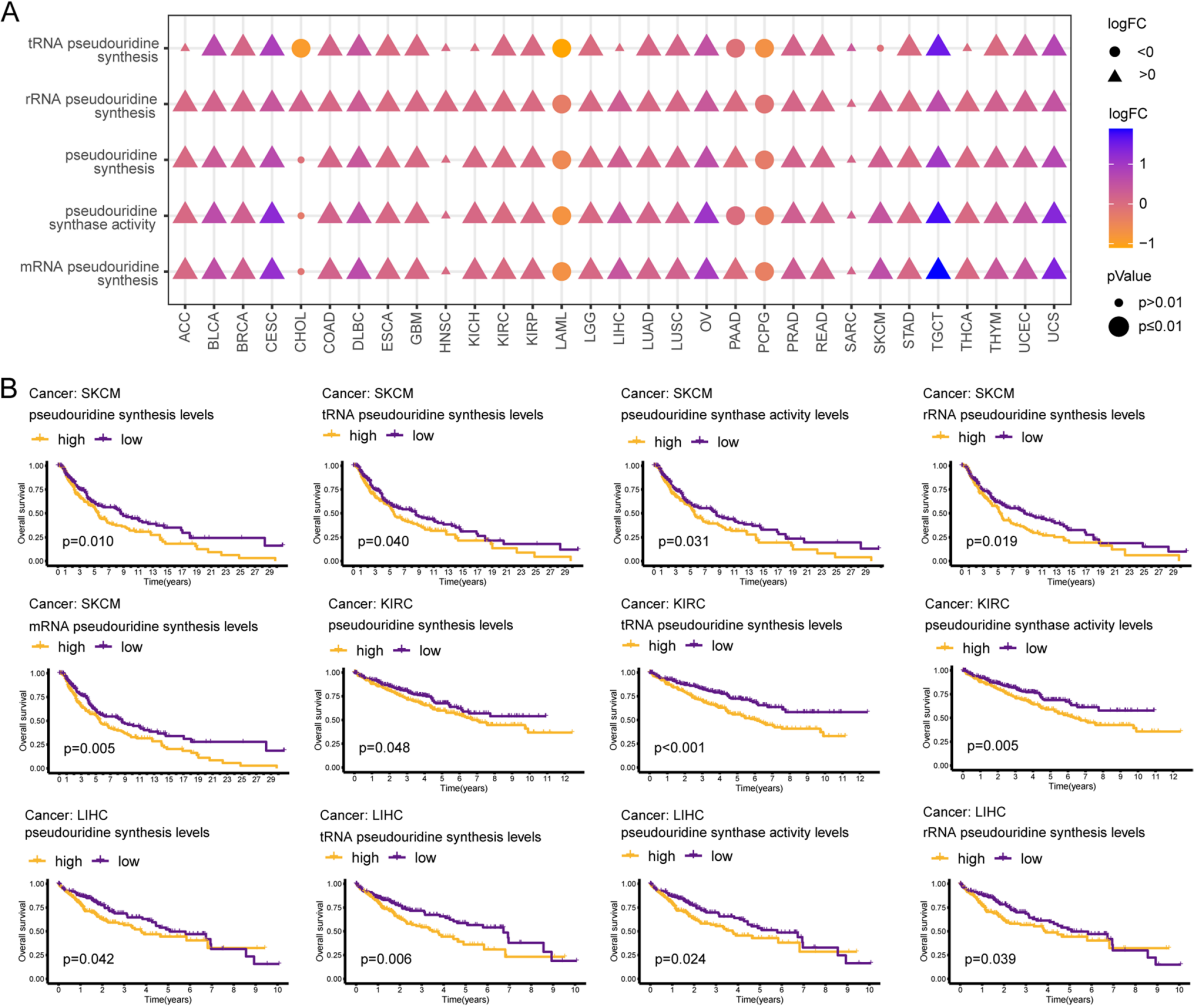
Abnormal pseudouridine processes. **A** Differential results of ssGSEA scores for five pseudouridine-related pathways in 31 tumor and nontumor tissues. Circles represent high ssGSEA scores for nontumors, and triangles represent high scores for tumors. **B** K‒M survival analysis of SKCM, KIRC, and LIHC tumors stratified by the median ssGSEA score

In this study, we downloaded the set of genes associated with pseudouridine processes from MSigDB and quantified the degree of activation of these processes using the ssGSEA algorithm. Abnormalities in five pathways, including rRNA pseudouridine synthesis, pseudouridine synthase activity, pseudouridine synthesis, mRNA pseudouridine synthesis, and tRNA pseudouridine synthesis, were observed in 30, 28, 28, 28, and 24 of 31 tumors (MSEO and UVM without control tissues), respectively, compared to nontumor tissues. The ssGSEA scores of these five pathways showed a significant increase, suggesting aberrant activation of these pathways, in 20 tumor types, including glioma, lung, and CRC. However, these processes were reversed in LAML and PCPG (Fig. 1A). Interestingly, a low activation state of all five pseudouridine-related processes was associated with an OS benefit in SKCM patients (p < 0.05). KIRC patients with consistently reduced activity of pathways for pseudouridine synthesis, pseudouridine synthase activity, and tRNA pseudouridine synthesis had a better prognosis (p < 0.05). LIHC patients with high levels of pseudouridine synthase activity, tRNA pseudouridine synthesis, rRNA pseudouridine synthesis, and pseudouridine synthesis had shorter OS (p < 0.05). In addition, these pathways were also found to be associated with OS in BRCA, LGG, LUAD, LUSC, PAAD, and SARC (p < 0.05) (Fig. 1B and S1A-G). In conclusion, pseudouridine-associated processes are aberrant in tumors and affect OS in patients with tumors. These abnormalities piqued our interest, and subsequent analyses were performed to explore the specific genes enabling them.

### Altered expression patterns of PUSs

**Fig. 2:**
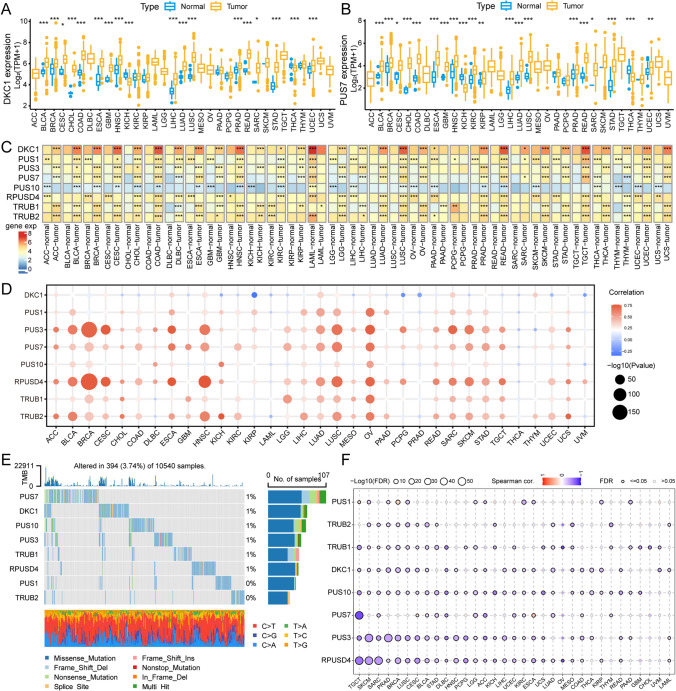
Characterizing the expression patterns of PUSs. **A**, **B** Differential expression of DKC1 and PUS7 (log_2_ (TPM + 1)) in tumors based on the TCGA dataset. Blue represents gene expression values in the normal group, and orange represents expression values in tumor tissues. **C** Each small square of the heatmap represents the median expression values of PUSs (log_2_ (TPM + 1)) in tumor and nontumor tissues (including TCGA and GTEx data). The square where * is located represents high and significantly different gene expression of that type. **D** Spearman correlation analysis of the expression of PUSs with corresponding CNV variants. The colors represent the degree of negative and positive correlation, respectively. The size represents log_10_ (p-value). **E** Waterfalls showing the mutations of eight PUSs across cancers. **F** Correlation analysis of the expression of PUSs with the degree of DNA methylation. The color changes represent the degree of correlation. The size represents the FDR value. The gray color indicates an FDR > 0.05. * p < 0.05; ** p < 0.01; *** p < 0.001

To explore the possible reasons for abnormal pseudouridine processes, we investigated the key enzymes regulating pseudouridine activity. The TCGA database comprises data on 24 tumor types with paired normal tissue. We observed varying degrees of expression increase or decrease in eight PUSs in more than 11 tumours. Most notably, PUS7 was aberrantly expressed in 20 tumor types (83%), with 75% of the tumors exhibiting upregulated PUS7 expression. Both PUS1 and TRUB2 were aberrantly expressed in 79% of the tumor types. Notably, the expression of all eight genes was significantly increased in BRCA, HNSC, KICH, LUSC, and STAD (Fig. 2A, B, and S2A-G). To elucidate the changes in the expression patterns of the regulators, we explored the differential mapping of these PUSs in 31 cancer types (MSEO and UVM did not have control tissue data) in conjunction with the GTEx database. According to the heatmap, at least two regulators are abnormally expressed in each tumor. The most notable aberrantly expressed genes were DKC1 and TRUB1, whose expression differed in 29 of the 31 cancer types. Overall, expression of all PUSs remained elevated in CHOL, COAD, LUSC, and READ tumor tissues. In contrast, in LAML tumors, the expression of seven of the eight genes was significantly lower, with only TRUB1 showing higher expression. In conclusion, synthetases involved in the pseudouridine process were aberrantly expressed in almost all cancer types, contributing to the dysregulation of this RNA modification process (Fig. 2C).

### Genomic aberrations of PUSs

Alterations in genetic information cause irreversible tumor development. Gene expression landscapes are often associated with copy number variation (CNV) [29]. We found that PUS1, PUS7, and PUS10 maintained consistent copy number amplification in most tumor types. In contrast, CNV deletions in the genes TRUB1 and RPUSD4 were maintained in most tumor types (Fig. S3A). Correlation analysis showed that CNV amplification was associated with high expression of most PUSs. In particular, the altered expression of PUS7 was attributed to CNV amplification, but the expression of DKC1 appeared to be less affected by CNV variation (Fig. 2D).

In addition, we explored mutations in pseudouridine-regulated proteases. Mutations were present in 394 of 10,540 samples from 33 tumors, with the most patients (107) having mutations in the PUS7 gene (Fig. 2E). In UCEC patients, the prevalence of PUS7 mutations reached 5%, and that of TRUB2 reached a minimum of 1% (Fig. S3B). Mutation types of all genes were dominated by missense mutations.

During tumorigenesis, key oncogenes are regulated by DNA promoter methylation, and aberrant methylation leads to the silencing of gene expression [30]. By comparing cancer tissues with normal tissues (tumor types with more than 10 associated normal tissues in the TCGA database), we found that the majority of PUSs showed dysfunctional methylation levels in tumors. Among them, DKC1 was significantly hypomethylated in several cancer tissues, including LIHC, HNSC, UCEC, PRAD, LUSC, THCA, BLCA, BRCA, and COAD (Fig. S3C). There is a clear and close link between aberrant methylation and the expression of pseudouridine regulatory factors, whereby the majority of PUS expression is negatively regulated by the degree of methylation. Notably, DKC1 promoter methylation was strongly associated with DKC1 mRNA expression in 24 tumor types (Fig. 2F). In contrast to the effect of CNV variants, DKC1 expression tended to be more affected by aberrant methylation.

### Correlation between PUSs expression and prognosis of tumor patients

**Fig. 3:**
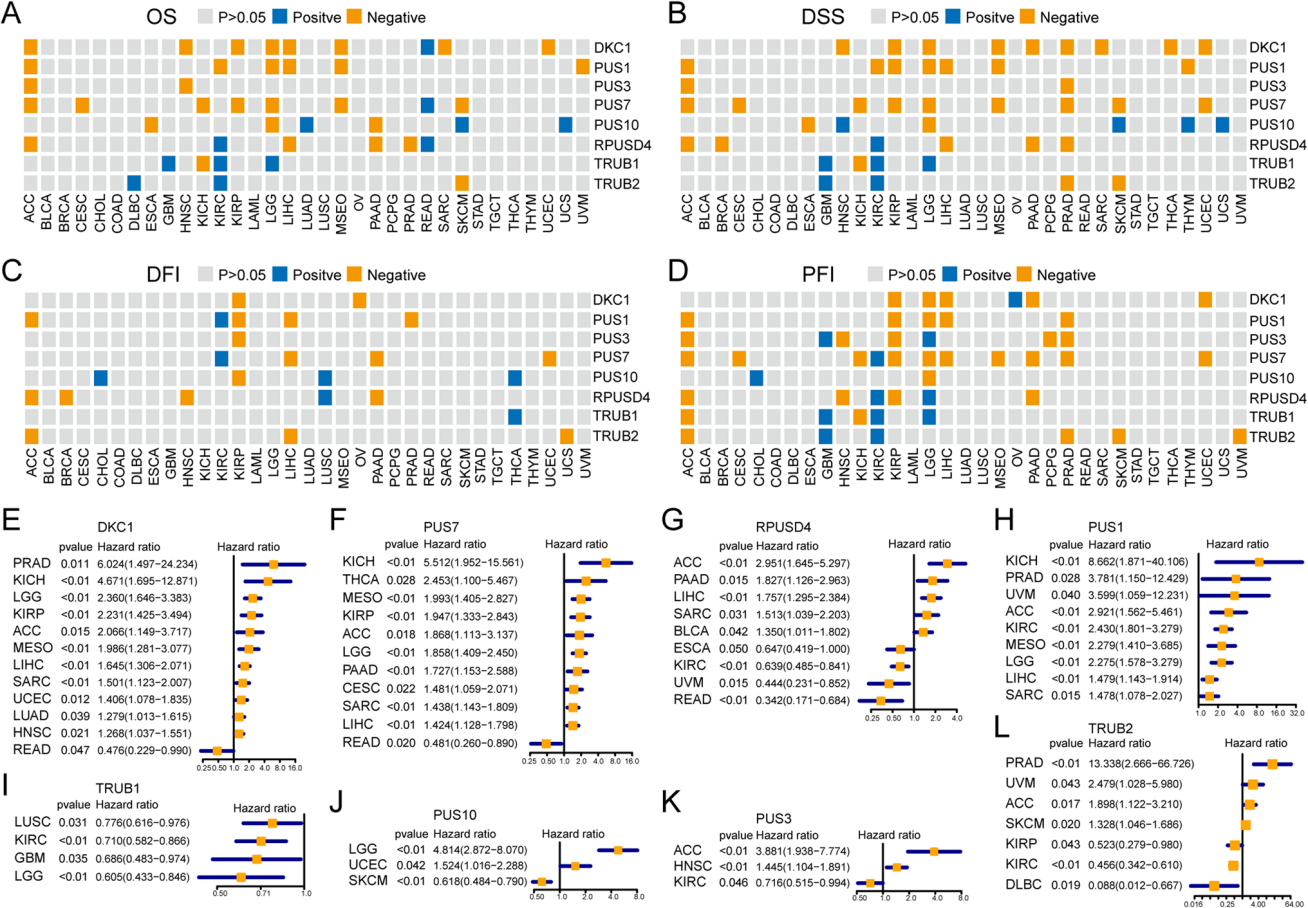
Prognostic value of PUSs in tumors. **A**–**D** Heatmap depicting the Kaplan‒Meier curve results for each PUSs across cancers. A gray color indicates p > 0.05, with a worse prognosis defined as “negative” as mRNA expression increases, and vice versa defined as “positive”. **A** shows overall survival (OS). **B** shows disease-specific survival (DSS). **C** shows the disease-free interval (DFI). The graph in **D** shows a progression-free interval (PFI). **E**–**L** Univariate Cox analysis showed the prognostic correlation of PUSs expression in multiple cancer types. The forest plot shows only the results with a p-value < 0.05

To elucidate the impact of aberrantly expressed PUSs on tumor prognosis, we performed a K‒M survival analysis. The expression of PUSs in 21 out of 33 tumor types correlated with OS; of these types, reduced expression of PUSs was favorable for OS in ACC, CESC, ESCA, HNSC, KICH, KIRP, LIHC, MESO, PAAD, PRAD, SARC, UCEC, and UVM tumors (Fig. 3A). The disease-specific survival (DSS) of patients with an additional 20 tumors types was affected by the expression of PUSs. The most affected tumor type was PRAD, in which the expression of five synthases, DKC1, PUS3, RPUSD4, PUS7, and TRUB2, had mostly negative effects on DSS. In addition, DKC1 and PUS7 expression were negatively correlated with DSS time in nine of the tumor types (Fig. 3B). In contrast, the disease-free interval (DFI) of the tumor types appeared to be the least affected. However, PUSs also maintained significant values in 14 tumors. Among these tumor types, increased expression of more than two PUSs negatively affected the DFI in ACC, KIRP, LIHC, and PAAD patients but positively affected the DFI in CHOL, KIRC, LUSC, and THCA patients (Fig. 3C). In ACC, KIRP, and PRAD, the expression of more than 4 genes negatively regulated the progression-free interval (PFI) in patients with tumors. PUS7 expression affected the PFI in more than 10 tumor types (Fig. 3D). Notably, OS, DSS, and PFI, but not the DFI, were modulated by PUSs expression in LGG. Furthermore, high TRUB1 expression tended to result in better OS, DSS, and PFI in both LGG and GBM. In contrast to other PUSs, TRUB1 seems to maintain a role in the prognoses of gliomas. This may be because TRUB1 expression specifically enhances the maturation of members of the let-7 family [31]. TRUB1, a highly conserved pseudouridine synthase, plays an important role in modifying the structure and function of mitochondrial tRNAs at position 55. In addition, let-7 can mediate posttranscriptional gene silencing to regulate growth inhibition in tumor cells. This could be a potential reason why TRUB1 is highly expressed and has prognostic value in GBM and LGG. Interestingly, the OS, DSS, DFI, and PFI of ACC, KIRP, and LIHC patients were affected by PUSs-regulated expression.

Subsequently, univariate Cox regression analyses revealed that the expression of DKC1 could serve as an independent risk element for PRAD, KICH, LGG, KIRP, ACC, MESO, LIHC, SARC, UCEC, LUAD, and HNSC tumor progression (Fig. 3E). PUS7 acted as an independent risk factor for ACC, CESC, KICH, THCA, MESO, KIRP, LGG, PAAD, SARC, and LIHC (Fig. 3F). PRUSD4 is a risk predictor for ACC, BLCA, LIHC, SARC, and PAAD tumors. Conversely, PRUSD4, DKC1, and PUS7 are also prognostic protective agents for READ. Along with TRUB1, TRUB2, and PUS3 expression, PRUSD4 was a protective factor for KIRC patients (Fig. 3G). In contrast, PUS1 expression, when used as an independent risk factor, affected only the development of ACC, KICH, PRAD, KIRC, MESO, LGG, LIHC, SARC, and UVM tumors (Fig. 3H). In contrast, TRUB1 expression had a protective effect only on tumors (Fig. 3I). PUS10 expression could act as a risk factor for LGG and UCEC and a protectant for SKCM patients (Fig. 3J). Increased PUS3 expression suggested a worse prognosis for ACC and HNSC (Fig. 3K). The dual predictive ability of TRUB2 expression was demonstrated by its ability to positively predict DLBC and KIRP, and to be a risk factor affecting the prognosis of patients with ACC, PRAD, and UVM (Fig. 3L). These results suggest that the eight proteases regulating pseudouridine are closely related to the prognoses of patients with tumors.

### PUSs and signaling pathways

**Fig. 4:**
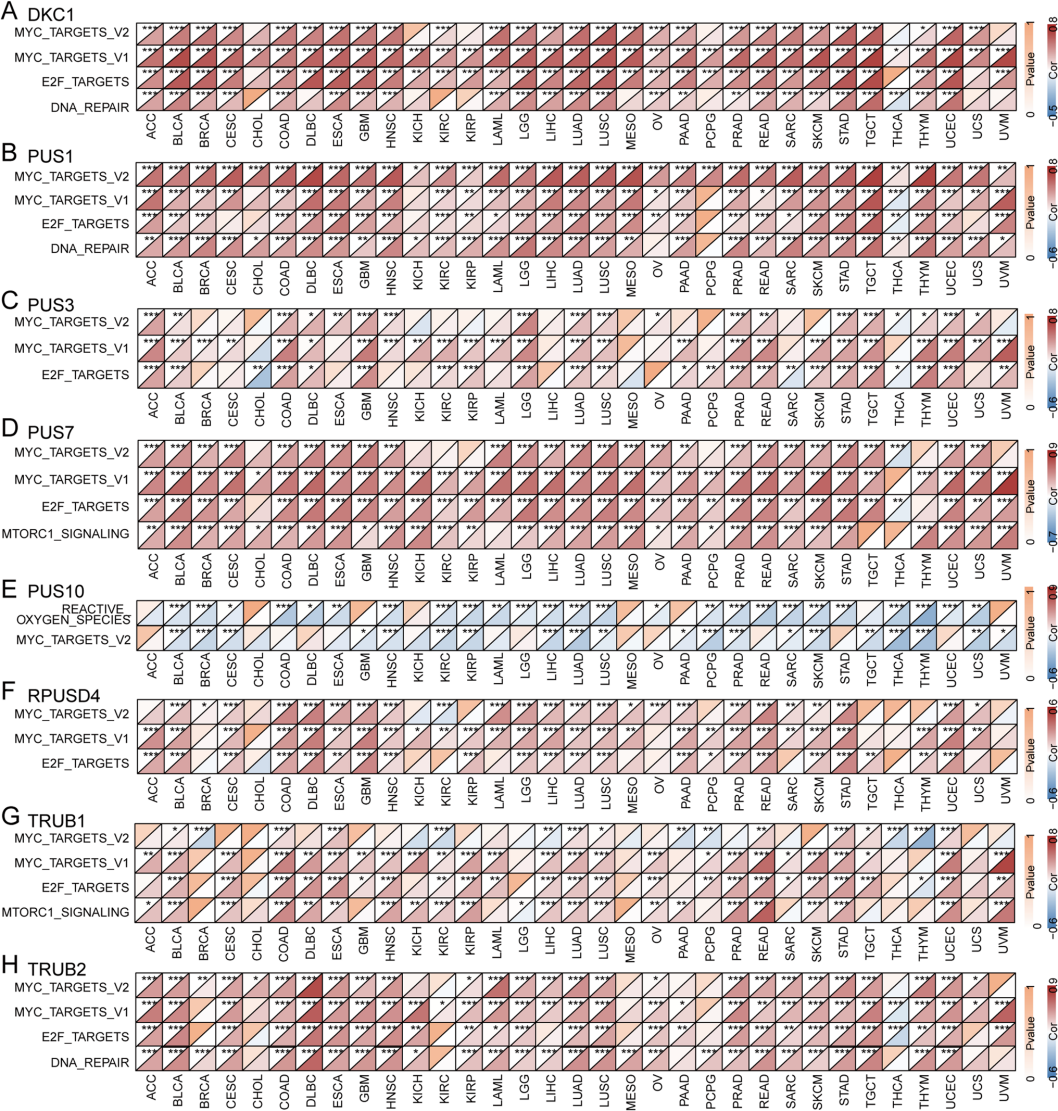
PUSs and signaling pathways. The activation scores of the MSigDB signature pathways were characterized by the ssGSEA algorithm. **A**–**H** Spearman correlation analysis was performed to determine the degree of association of PUSs expression with the ssGSEA scores of those pathways. The heatmaps show pathways with commonalities. Triangles above and below each heatmap square indicate significance and correlation, respectively. The threshold was a p value < 0.05 (* p < 0.05; ** p < 0.01; *** p < 0.001)

Abnormally expressed genes often originate from or result in oncogenic events. To explore the effects of PUSs expression on signaling pathways, we calculated 50 characteristic pathway enrichment scores for 33 tumors via the ssGSEA algorithm. Spearman correlation analysis revealed that the expression of all seven PUSs was robustly and positively correlated with the ssGSEA enrichment scores of MYC-TARGETS_V1, MYC_TARGETS_V2, E2F_TARGETS, and MTORC1 in more than 20 tumors. In all tumor types, the expression of DKC1 was strongly correlated with MYC-TARGETS_V1 activation. The expression of PUS1 also maintained a stable positive correlation with MYC_TARGETS_V2 enrichment. However, the expression pattern of PUS10 differed from that of other regulators in that it showed a stable negative correlation with enrichment of MYC_TARGETS_V2, which was validated in 20 tumor types (Fig. 4A–H). The enriched fractions of MYC_TARGETS_V1, MYC_TARGETS_V2, E2F_TARGETS, and MTORC1 were significantly greater in tumors than in compared to normal tissues (Fig. S4). We speculate that the aberrant activation of these pathways may be related to the aberrant regulation of PUSs.

### PUSs modulate the TME

**Fig. 5:**
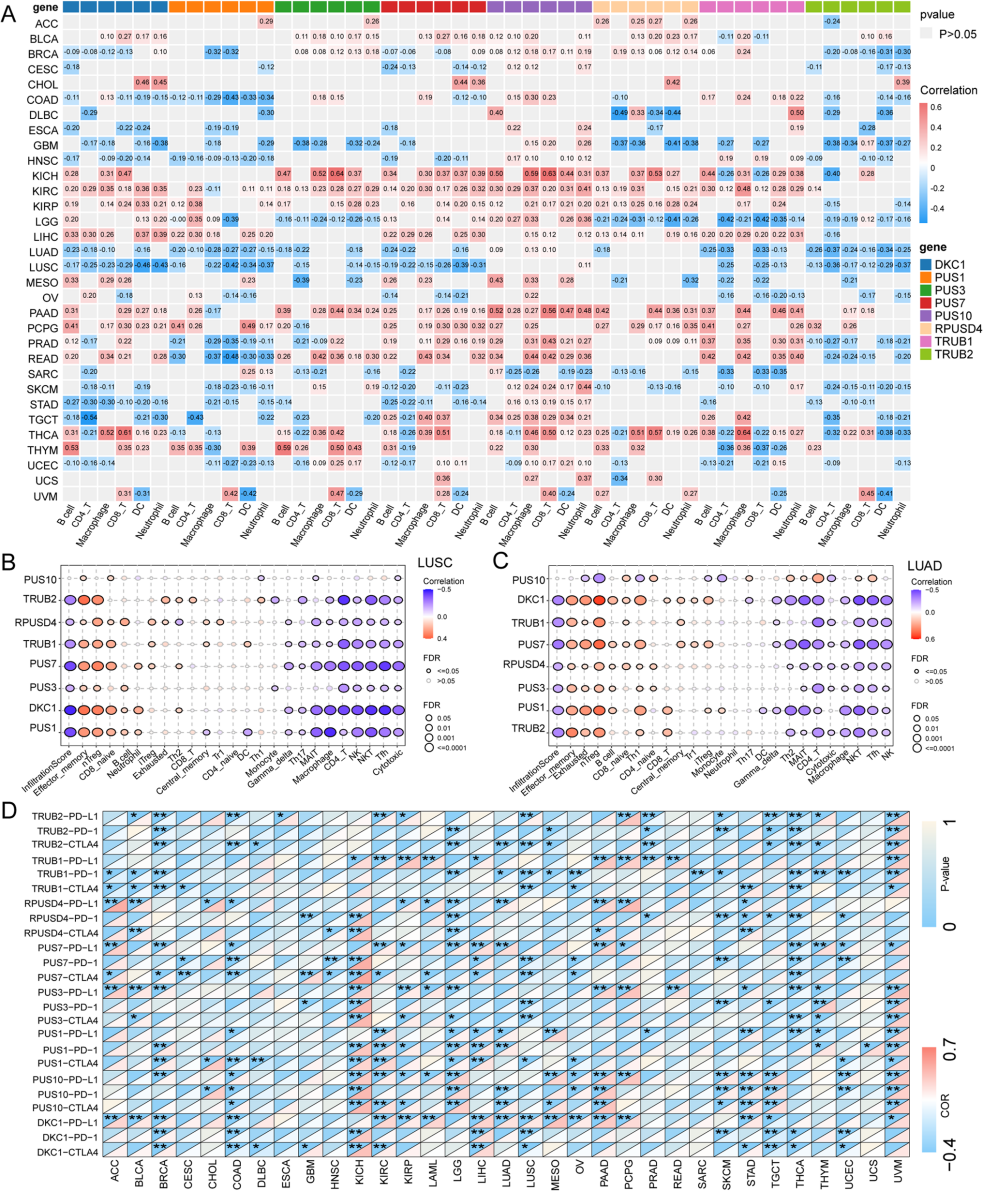
PUSs are involved in the regulating of TME. **A** Correlations of the abundances of infiltrating B-cells, CD4 + T-cells, macrophages, CD8 + T-cells, dendritic cells (DCs), and neutrophils with the expression of PUSs in 33 cancer types based on the TIMER algorithm. The gray color represents p > 0.05. Specific values in the heatmap represent Spearman correlation coefficients. **B**, **C** Bubble plots demonstrating the correlation between PUSs expression in LUSC (**B**) and LUAD (**C**) and immune cell infiltration. The threshold was an FDR <  = 0.05. The colors indicate the magnitudes of the positive and negative correlations. **D** Heatmap showing the degree of correlation between PUSs expression and ICPs. The upper and lower triangles in each heatmap square indicate significance and correlation, respectively. The threshold for significance was p < 0.05 (* p < 0.05; ** p < 0.01). The abbreviations in the figure correspond to NK, NK cell; NKT, natural killer T cell; Gamma_delta, gamma delta T cell; CD4_naive, CD4 naïve T-cell; CD8_naive, CD8 naïve T-cell; Cytotoxic, cytotoxic T-cell; Exhausted, exhausted T-cell; Tr1, type 1 regulatory T cell; nTreg, natural regulatory T cell; iTreg, induced regulatory T cell, Th1, T helper type 1; Th2, T helper type 2; Th17, T helper type 17; Tfh, T follicular helper cell; Central_memory, central memory T-cell; Effector_memory, effector memory T-cell; MAIT, mucosal associated invariant T-cell; Infiltration Score, Overall infiltration score of 24 immune cells

The potential link between RNA modification and immune infiltration has gradually attracted increasing attention [32]. The TIMER algorithm was used to estimate the infiltration of six immune cell types in tumor tissues using RNA-seq expression profiles of from the TCGA. We calculated the correlation between PUSs expression and the infiltration of these immune cells. Overall, we observed that PUSs expression was mainly involved in immune infiltration in solid tumors. For example, in kidney cancer types including KICH, KIRC, and KIRP, the expression of most PUSs, was significantly positively correlated with the six immune cell types. In lung cancer (LUAD, LUSC), DKC1, PUS1, PUS3, PUS7, TRUB1, and TRUB2 expression was mostly negatively correlated with immune cell infiltration. However, PUS3, PUS10, TRUB1, and TRUB2 expression maintained similar correlations with immune cell infiltration in GBM and LGG. In addition, high expression of PUS7 and PUS10 in PAAD, PCPG, PRAD, READ, TGCT, and THCA tumors was associated with increased B-cell and macrophage infiltration. Interestingly, in UVM, increased expression of DKC1, PUS1, PUS3, PUS7, PUS10, and TRUB2 was significantly correlated with increased CD8 + cell infiltration and decreased DC infiltration (Fig. 5A).

Given the potential association of PUSs with the TME in lung cancer (LUAD and LUSC), we focused on the immune landscapes of these lung cancers. An algorithm based on ssGSEA, which has a more detailed classification of immune cell infiltration, produced results that were similar to those of the TIMER algorithm. Overall, the expression of PUSs was negatively correlated with the total infiltration score in LUAD and LUSC. This score was positively correlated with the number of immunosuppressive nTregs and negatively correlated with the infiltration scores of NK and CD4 + T-cells via tumor-killing effects. Increased expression of pseudouridine modulates the immunosuppressive TME (Fig. 5B, C). Furthermore, in KIRC, we found that PUS1, PUS10, PUS7, and RPUSD4 expression showed robust positive correlations with overall infiltration scores and stable positive correlations with the infiltration of a variety of immune cells, including nTregs, iTregs, macrophages, Th1 cells, monocytes, and Tfh cells. TRUB1 and TRUB2 expression maintained a stable negative correlation with immune cell infiltration. This trend seems to be opposite to that calculated by the TIMER algorithm, which may be related to the way the algorithm is calculated and interpreted. Overall, however, there is a potential correlation between PUSs expression and immune cell infiltration. This provides a referenceable direction for researchers studying pseudouridine in the future (Fig. S3D).

With this in mind, we explored the effect of PUSs expression on the expression of ICPs. We found that in renal cancer, the expression of PUSs significantly affects CD8 + and CD4 + T-cell infiltration. However, this increase was accompanied by an increase in protease expression, with a steady increase in the expression of PD-1, PD-1, and CTLA4. This may limit the killing effect of these immune cells. This phenomenon was also observed in UVM, PAAD, and PCPG tumors (Fig. 5D).

### Construction of the pseudouridine signature score

**Fig. 6:**
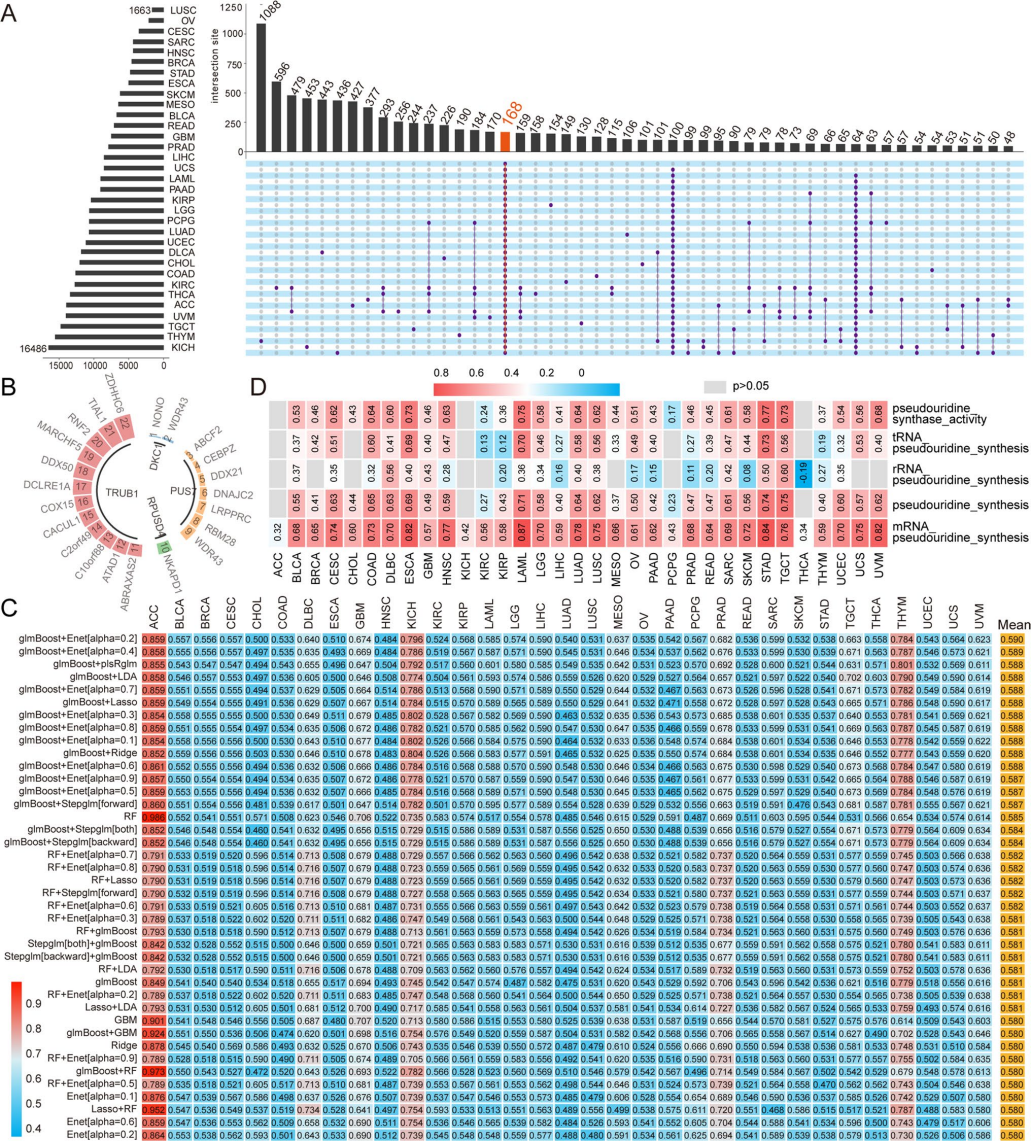
Construction of the pseudouridine signature score. **A** UpSet plots across tumors generated an intersection plot of genes associated with PUSs, and the top 50 genes are shown. **B** Twenty-one genes with corresponding PUSs. The outer circle represents 21 of the 168 genes (WDR43 repeats) and the inner circle represents 4 of the PUSs. **C** Construction of pseudouridine feature models using a framework of 112 machine learning algorithms in different combinations, with ACC tumor as the training echelon and other tumor datasets as the validation cohort. The heatmap demonstrates the prognostic C-index of the feature model in the tumor (TP40). **D** Correlation analysis of the pseudouridine signature score with the processes associated with pseudouridine. The correlation coefficients are labeled with specific values. Gray and no values represent low significance (p > 0.05)

To better describe the degree of pseudouridine modification within a tumor, we constructed a pseudouridine feature score. Facing statistical methodological difficulties in modeling high-dimensional data, we applied the integration of multiple machine-learning modeling strategies to improve the accuracy and robustness of the feature model score. First, we performed Pearson correlation analysis of all genes in 33 tumors with the expression of eight PUSs to screen genes with potential interactions. Upset plots showed that the expression of 168 genes was strongly associated with that of PUSs in all tumor types (Fig. 6A). Moreover, the expression correlations of 21 of the 168 genes with the corresponding PUSs were consistent across all tumors (Fig. 6B). Based on the expression of these 21 genes and 8 PUSs, we trained 112 prognostic models for each tumor type using an aggregated machine learning approach and applied them to other tumors. The prognostic power of each model for tumors was assessed based on the average C-index. Ultimately, we found that the ACC-based genes screened with glmBoost + Enet[alpha = 0.2] had the highest average C index (0.590). We used this as a template to construct a prognostic model and applied it to ACC, DLBC, GBM, KICH, MESO, THYM, TGCT, and PRAD tumors with good prognostic ability (C-index > 0.6) (Fig. 6C and S5A).

The pseudouridine signature scores we constructed correlated with the mRNA pseudouridine synthesis pathway in all tumors (p < 0.05). The tumor with the strongest correlation was LAML, with a correlation coefficient of 0.87. In addition, the pseudouridine signature score correlated with the pseudouridine synthase activity and pseudouridine synthesis ssGSEA scores in 30 tumors, i.e., the signature model also better described these two functional processes in tumors (Fig. 6D).

### Predictive chemotherapy drugs

**Fig. 7:**
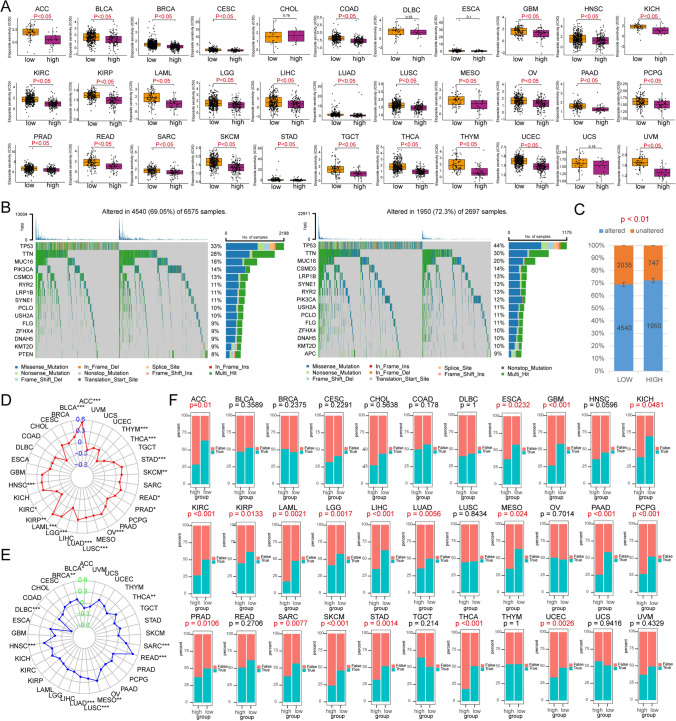
Prediction of antitumor therapy response. **A** The IC50 values of the chemotherapeutic agent etoposide predicted by the ‘pRRophetic’ method were different between groups in different tumors. **B** Waterfall plots showing differences in the mutation status between the two groups with low and high signature scores across cancers. **C** Stacked plots showing the distribution of mutation characteristics between the high- and low-score groups. P values are from Fisher’s exact test. Radar plots indicating the strength of the association of signature scores with **D** TMB or **E** MSI. **F** Stacked plots showing 33 tumors exhibiting the characteristic distribution of ICB response between two groups with low- and high-signature scores. ICB responses were predicted by the TIDE algorithm. P values were obtained by Fisher’s exact test. * p < 0.05; ** p < 0.01; *** p < 0.001

We divided the different tumor samples into high-signature score and low-signature score groups based on the pseudouridine signature score. We found that the signature score showed a significant association to the therapeutic effect of etoposide. Across 29 tumors, the IC50 of etoposide in the high-signature score group was much lower than that in the low-signature score group, implying that the high group was more dependent on etoposide treatment (Fig. 7A). In addition, two drugs, camptothecin and methotrexate, were more suitable for the treatment of patients with BLCA, BRCA, LAML, KIRC, HNSC, GBM, LGG, LUAD, SKCM, SARC, THYM, UCSC, and UVM tumors with high pseudouridine status (Figs. S6 and S7). Low-scoring BLCA, BRCA, HNSC, KIRP, LGG, PAAD, SARC, SKCM, TGCT, and UCSC were more tolerant to cisplatin (Fig. S8). The high subgroups of CESC, KIRC, MESO, PRAD, SARC, and THCA had lower IC50 values for the chemotherapeutic agent bexarotene and were more amenable to bexarotene treatment. The low subgroups LAML, LUAD, STAD, UVM, and THYM were more suitable for bexarotene treatment (Fig. S9). The above results suggest that the pseudouridine signature score has the potential to guide the selection of clinical chemotherapeutic agents for different tumors.

### Stratification analysis and mutation status

Analysis of tumor mutation information allows us to better understand patients with different characteristics. In addition, somatic mutation rates are often associated with tumor treatment. The mutation rate was 69.05% in the low signature-scoring group and 72.3% in the high-scoring group (p < 0.05). This implies that the higher-scoring group with a higher somatic mutation rate possesses a high release of tumor neoantigens and more potent anticancer immune activity [33, 34]. In addition, the gene with the highest mutation rate in both sample groups was TP53 (33% of the low-scoring group and 44% of the high-scoring group) (Fig. 7B, C). In contrast, the signature scores were significantly greater for the TP53-mutated ACC, BLCA, BRCA, COAD, HNSC, LIHC, LUAD, LUSC, PRAD, READ, SKCM, STAD, UCEC, and UCS samples (Fig. S5B).

TMB has emerged as a quantitative genomic biomarker of response to ICB [35]. We found that our constructed pseudouridine signature score was stably and positively correlated with TMB in ACC, BLCA, HNSC, LAML, LGG, LUAD, LUSC, OV, PRAD, READ, SKCM, STAD, THCA, and THYM tumors and negatively correlated with KIRC and KIRP tumors (Fig. 7D). This suggests a potential association between the characterization model and ICB treatment response. This conjecture has been confirmed in the exploration of MSI, which has emerged as a "predictive" biomarker of immunotherapy benefit [36]. We observed a stable positive correlation between pseudouridine scores and MSI in BRCA, BLCA, HNSC, LUAD, LUSC, MESO, READ, SARC, and THCA (Fig. 7E).

### Predictive ICB responsiveness

ICB has yielded surprising results in the treatment of a wide range of tumors, including melanoma and lung cancer [37]. Given our previous findings of a potential link between PUSs and ICP expression, we hypothesized that pseudouridine signature models may also have the ability to predict immunotherapy efficacy. We further explored this using the TIDE algorithm. This algorithm integrates a novel computational framework based on large-scale histological and biomarker data from published ICB trials to quantify tumor immune dysfunction and rejection based on gene expression profiles of tumor samples, and to further assess the likelihood of tumor immune escape and response to ICB. With this in mind, we performed a stratified predictive analysis of the pseudouridine signature model for ICB therapy. The results suggest that the pseudouridine signature model is a good predictor of the effect of immunotherapy on tumors. ACC, ESCA, GBM, KICH, KIRC, KIRP, LAML, LGG, LIHC, LUAD, MESO, PAAD, PCPG, PRAD, SARC, SKCM, STAD, THCA, and UCEC patients with lower pseudouridine scores were more sensitive to ICB therapy (Fig. 7F).

## Discussion

Cancer development and progression are multifactorial, and rely on mechanisms related to genomic variation and epigenetic imbalances [38]. Genetic and epigenetic abnormalities regulate gene expression in different tumors and broadly influence RNA synthesis, catabolism, expression, and consequently TME and drug resistance. Pseudouridine modification was one of the first identified and most abundant types of RNA modification associated with cancer [39]. Our studies of the pseudouridine-related pathway have shown that there is a clear abnormality in this modification process in tumors and that this abnormality affects the prognoses of tumor patients. Several researchers have detected high levels of pseudouridine in the body fluids of patients with colon, prostate, and ovarian cancers [40]. In addition, abnormalities in pseudouridine modification have been found to predict disease progression in several leukaemia studies [41, 42]. In contrast, our study revealed that the activation of pathways related to pseudouridine modification appeared to be elevated in control tissues of LAML patients. This may be due to the small sample size of the control group. In addition, after removing the batch effect, we considered that the RNA-seq data sources for LAML and control tissues were different and not fully compatible. In conclusion, these results suggest that the modification of pseudouridine may be a potential mechanism for cancer development and progression and that this modifier could serve as a biomarker. There is growing interest in the role of such RNA modifications and modifying enzymes in cancer. However, apart from the fact that most studies have focused on the effects of a single molecule or a single tumor, few studies have explored the common role of pseudouridine in multiple tumors. To fill this scientific gap, our study integrates data from patients with all tumors in the TCGA database and confirms the importance of PUSs expression in cancer bioregulation. The mechanistic details of pseudouridine formation remain controversial, but all of the potential mechanisms rely on the activities of regulatory proteases. Previous studies have focused on the mechanism of action of individual regulators. DKC1 expression is upregulated in CRC and stabilizes the mRNAs of several ribosomal proteins, thereby promoting cancer progression. In addition, inhibition of DKC1 abrogates the inhibitory effect of ribosomal proteins on HRAS and induces activation of the oncogenic RAS/RAF/MEK/ERK pathway [10]. In addition, inhibition of DKC1 induced telomere-associated senescence and apoptosis in lung adenocarcinoma cells [43]. PUS1 expression knockdown significantly affects a range of cancer-related biological processes, such as the regulation of cell proliferation and cell migration, mitochondrial autophagy, and PI3K/Akt signaling [44]. PUS7 expression promotes CRC cell proliferation by activating the Wnt/β pathway through direct stabilization of SIRT1. Knockdown of PUS7 inhibits the proliferation of CRC cells in vitro [45]. Expression of TRUB1, which is an evolutionarily conserved microRNA that mediates posttranscriptional gene silencing and regulates a variety of biological processes, including development, differentiation, and tumor suppression, promotes pseudouridine installation and let-7 maturation in human tumor cells [31]. However, less attention has been given to the relationship between the expression of these PUSs and CNV alterations and DNA methylation. Our findings suggest that two factors, DKC1 and PUS7, have representative characteristics. First, both enzymes are aberrantly expressed in tumors and affect patient prognosis. However, the expression of PUS7 seems to be associated with CNV alterations, whereas abnormalities in DKC1 expression seem to be more regulated by DNA methylation. Further researches on the specific underlying mechanisms are needed. Our study provides a new perspective for a wide range of scholars studying this unique and important modification pattern. Even for regulators with the same role, their expression patterns are not always the same.

Regarding the exploration of potential functions and signaling pathways affected by pseudouridine modification regulators, we found that these dysregulated genes were associated mainly with MYC-related oncogenic pathways. MYC plays a regulatory role in a variety of cellular processes, such as proliferation, differentiation, and apoptosis [46]. In neuroblastoma, both N-Myc and C-Myc have been reported to directly target the DKC1 promoter, resulting in elevated DKC1 mRNA expression levels [47]. High DKC1 expression independently predicts PFS in female patients, and DKC1-overexpressing tumors show more frequent alterations in the PIK3CA, MYC, and TP53 genes [48]. MYC was significantly regulated in colon cancer cells with restricted or enhanced expression of PUS7. Our results are similar to these reports, and we also found that MYC is not only associated with DKC1 and PUS7 but also involved in the dysregulation of other PUSs. The reasons for the disordered expression of PUSs are multifaceted, and in addition to methylation and CNV alterations, key oncogenic pathways may also be important links. Our findings complement the potential impact of the oncogenic process of MYC on RNA modification and provide new ideas for exploring the major driving model factors for the dysregulation of pseudouridine modification in human tumorigenesis. In addition, we found a robust correlation between E2F expression and the regulatory factor pseudouridine. The transcriptional activity of E2F is of significant value for cellular homeostasis. Overactivation of E2F promotes malignant proliferation of otherwise quiescent cells [49]. In addition, Myc proteins can also induce the expression of genes encoding the E2F1, E2F2, and E2F3 transcription factor proteins. Taken together with our results, these findings indicate that aberrant cell cycle activity and malignant proliferation of tumor cells crosstalk between PUSs, E2F, and MYC. Notably, the specific role that aberrantly accumulating pseudouridine modifications play in the imbalance between MYC and E2F warrants further exploration. As the most abundant RNA modification in cells, the upstream and downstream events of pseudouridine abnormalities, which will facilitate research into the origins of tumor development and metastatic mechanisms, are worth revealing.

The TME also plays a crucial role in tumor proliferation, metastasis, and treatment resistance. Therefore, we also investigated the link between pseudouridine modifications and the TME. The correlations between PUSs expression and immune cell infiltration, as well as ICPs expression, suggest that PUSs may be potential factors in shaping the TME. This finding is similar to the results of several bioinformatics studies [9, 50, 51]. Undeniably, B cells seemed to show the opposite trend in our study. We believe this may be related to the use of two different algorithms. Gene expression profiling using RNA-seq is an effective method for determining the abundance of immune cells. Existing computational algorithms for immune infiltration estimation can be divided into two broad categories: feature gene set-based methods and deconvolutional statistical-mathematical methods [52]. The former method utilizes feature genes from different cell types, which are then enriched and analyzed by the expression values of the feature genes in the tissue samples to infer the enriched transcriptome profiles in the tissues [53, 54]. The inverse convolution method defines the problem as a mathematical equation that models gene expression in tissue samples as a weighting of cell expression profiles in a population mixture [55–57]. These two complementary algorithms show different performance advantages in estimating specific immune cell types for different tumors. Figure 5A shows the reverse convolution of the TIMER algorithm [21], while the data presented in Fig. 5B is based on feature genes followed by enrichment analysis. Notably, we focused on results with the same trends, such as increased CD8 + cell infiltration and decreased DC numbers. The same results from both algorithms may represent more convincing and rigorous evidence. In addition, PDL1, PD1, and CTLA4 expression are considered key components of antitumor immune surveillance. The expression of PDL1, PD1, and CTLA4 in kidney and lung cancers increased with increased expression of PUSs. We also found that in lung cancer, this effect was accompanied by increased Tregs infiltration. These results led to the phenomenon of antitumor immunosuppression by TME formation. However, given the complexity of the TME and the specificity of different tumors, the mechanisms by which pseudouridine shapes the TME and antitumor immune function need to be further explored.

Multiple mechanisms dominate the different prognoses of patients with tumors. In the absence of highly effective oncological treatments, it is worthwhile for clinicians to assess the risk of death and decide on an optimal treatment strategy to maximize the clinical benefit to patients. Among the barriers to treatment are limited data available for research and constraints in screening conditions. Inspired by the work of Liu et al., we used 112 fusion machine learning methods from which we selected the best prognostic model [23]. Prior to machine learning, we also identified genes with potential interactions with pseudouridine in large samples and multiple tumor types. This approach greatly improves the commonality and representativeness between pancancer sample datasets. Through multistep screening and coefficient determination by multiple fusion machine learning, we constructed a robust model, which was confirmed by system performance comparisons. This study provides a basis for accurate prognostic judgment. The combination of pyrazofuran (DKC1 inhibitor) and trametinib (MEK inhibitor) synergistically inhibits the growth of CRC cells [10]. This prompted us to study the use of prognostic models for pseudouridine to predict the efficacy of antitumor therapy. Overall, prognostic modeling was valuable for predicting chemotherapeutic drug sensitivity and immunotherapeutic response. Admittedly, the model performed well for only a few tumor types (most cancers had a C-index below 0.6). This may be due to the difficulty of using statistical methods to model high-dimensional data, although we used multiple machine learning modeling strategies to improve the accuracy and robustness of the feature model scores. The reason may also be that the gene expression underlying the constructed models does not necessarily have similar prognostic value in all tumors. The models we constructed were able to describe the pseudouridine signatures to some extent while having some predictive prognostic power. We referenced several studies to inform our strategy, but few algorithms can perfectly account for certain features in 33 tumors while retaining robust prognostic value. We will continue our exploration to accomplish this difficult task.

Our study confirmed the aberrant expression patterns, prognostic value, and potential functions of these regulators, further strengthening the concept of dysregulated pseudouridine modification in cancer. Nevertheless, certain limitations of our study must be acknowledged. First, in-depth proteomic analysis was lacking, which may have led to biased conclusions. Second, the specific molecular mechanism by which PUSs promote tumor proliferation has not been fully validated in vitro and in vivo. The predictive ability of prognostic models for chemotherapeutic agents also needs to be validated in many studies. This limits the results of our study. However, we will incorporate these studies in our future work.

## Conclusion

In conclusion, a comprehensive study of regulatory genes of pseudouridine modification across cancers revealed that the expression patterns of relevant PUSs are influenced by genomic aberrations and DNA methylation. Our exploration of the associations between pseudouridine and the TME and antitumor immunity provides valuable insights for future research. Of particular importance, our machine learning-based adjustment for potential biasing factors allowed our prognostic model to maintain valid prognostic metrics for large samples of multitype tumor data. Its excellent performance in predicting chemotherapy and immunotherapy response sensitivity was also demonstrated. These findings may provide new ideas for the use of pseudouridine as a potential pancancer therapeutic target.

### Supplementary Information

Supplementary file 1 (TIF 679 KB)

Supplementary file 2 (TIF 2480 KB)

Supplementary file 3 (TIF 2795 KB)

Supplementary file 4 (TIF 6297 KB)

Supplementary file 5 (TIF 8448 KB)

Supplementary file 6 (TIF 2710 KB)

Supplementary file 7 (TIF 2682 KB)

Supplementary file 8 (TIF 2682 KB)

Supplementary file 9 (TIF 2682 KB)

Supplementary file 10 (DOCX 21 KB)

Supplementary file 11 (DOCX 20 KB)

Supplementary file 12 (DOCX 21 KB)

## Data Availability

The data set for this study is available from public databases. See the Methods section of the manuscript for details. The datasets generated for this study are available on request to the corresponding author.

## Abbreviations

ACC: Adrenocortical cancer
BLCA: Bladder cancer
BRCA: Breast cancer
CESC: Cervical cancer
CHOL: Bile duct cancer
CNV: Copy number variation
COAD: Colon cancer
DFI: Disease-free interval
DLBC: Large B-cell lymphoma
DSS: Disease-specific survival
Enet: Elasticity network
ESCA: Esophageal cancer
GBM: Glioblastoma
GBM (Machine Learning): Generalized boosted regression model
HNSC: Head and neck cancer
ICB: Immune checkpoint blockade
ICPs: Immune checkpoints
KICH: Kidney chromophobe
KIRC: Kidney clear cell carcinoma
KIRP: Kidney papillary cell carcinoma
K‒M: Kaplan‒Meier
LAML: Acute myeloid leukemia
LGG: Lower grade glioma
LIHC: Liver cancer
LOOCV: Leave-one-out cross-validation
LUAD: Lung adenocarcinoma
LUSC: Lung squamous cell carcinoma
MESO: Mesothelioma
MSI: Microsatellite instability
MSigDB: Molecular signature database
OS: Overall survival
OV: Ovarian cancer
PAAD: Pancreatic cancer
PCPG: Pheochromocytoma & Paraganglioma
PFI: Progression-free interval
PRAD: Prostate cancer
PUSs: Pseudouridine synthases
READ: Rectal cancer
RF: Random forest
SARC: Sarcoma
SKCM: Skin cutaneous melanoma
STAD: Stomach cancer
TCGA: The cancer genome atlas
TGCT: Testicular cancer
THCA: Thyroid cancer
THYM: Thymoma
TIDE: Tumor immune dysfunction and rejection
TMB: Tumor mutation burden
TME: Tumor microenvironment
UCEC: Endometrioid cancer
UCS: Uterine carcinosarcoma
UCSC: University of California Santa Cruz
UVM: Uveal melanoma
