# Supplementary figures and images for: Multiomics and machine learning-based analysis of pancancer pseudouridine modifications
Source: Discov Oncol. 2024 Aug 20;15:361. doi: 10.1007/s12672-024-01093-y (PMC11335713; doi:10.1007/s12672-024-01093-y)

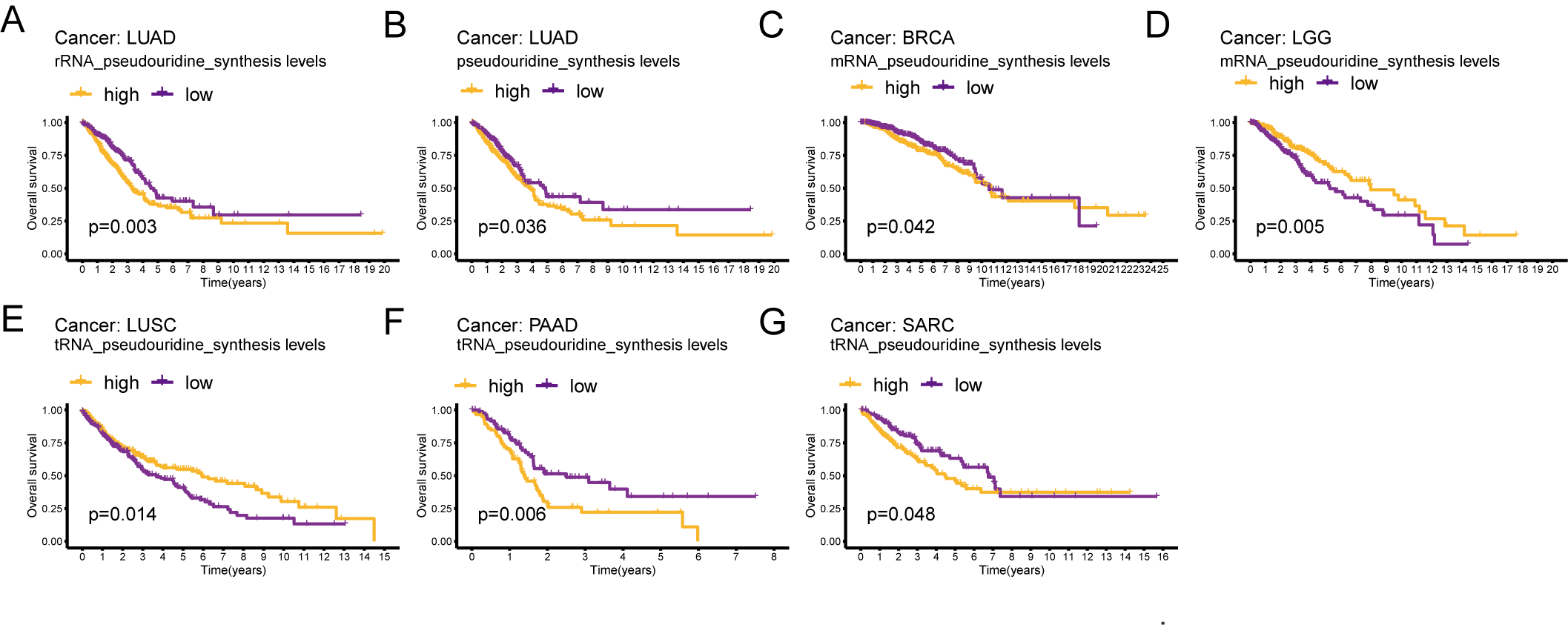

Supplement: Supplementary file 1 — (TIF 679 KB) [file 12672_2024_1093_MOESM1_ESM.tif]

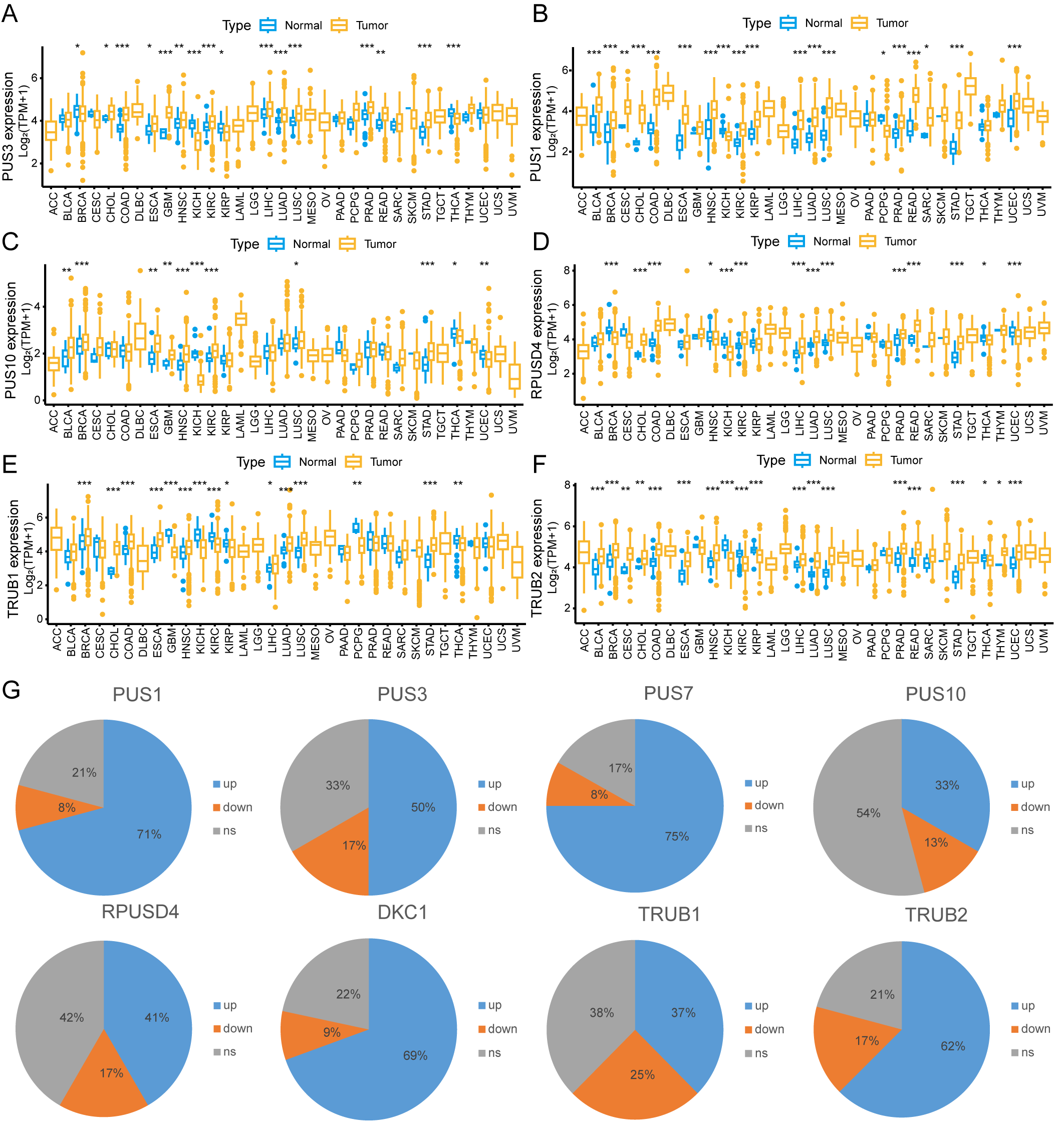

Supplement: Supplementary file 2 — (TIF 2480 KB) [file 12672_2024_1093_MOESM2_ESM.tif]

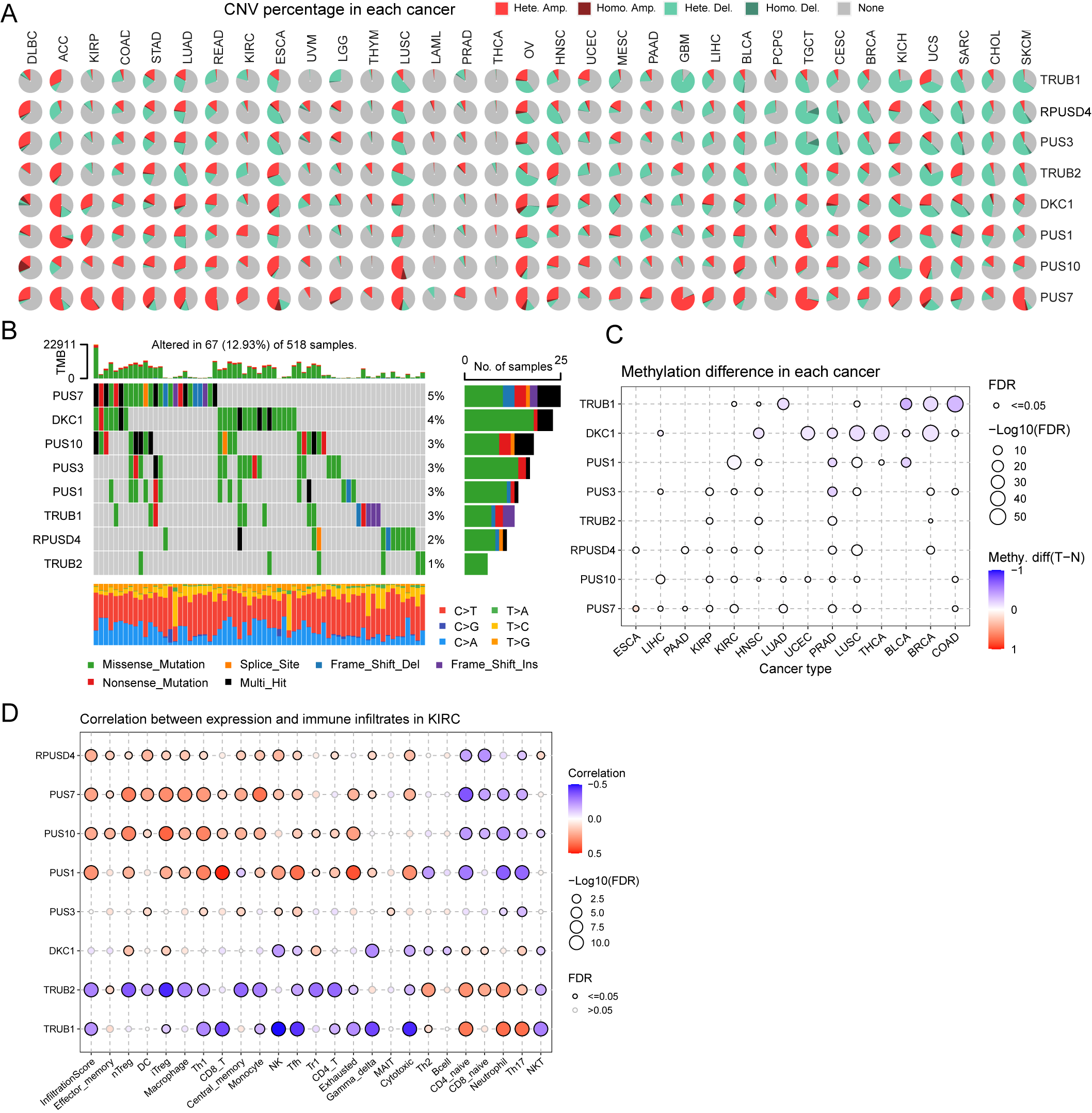

Supplement: Supplementary file 3 — (TIF 2795 KB) [file 12672_2024_1093_MOESM3_ESM.tif]

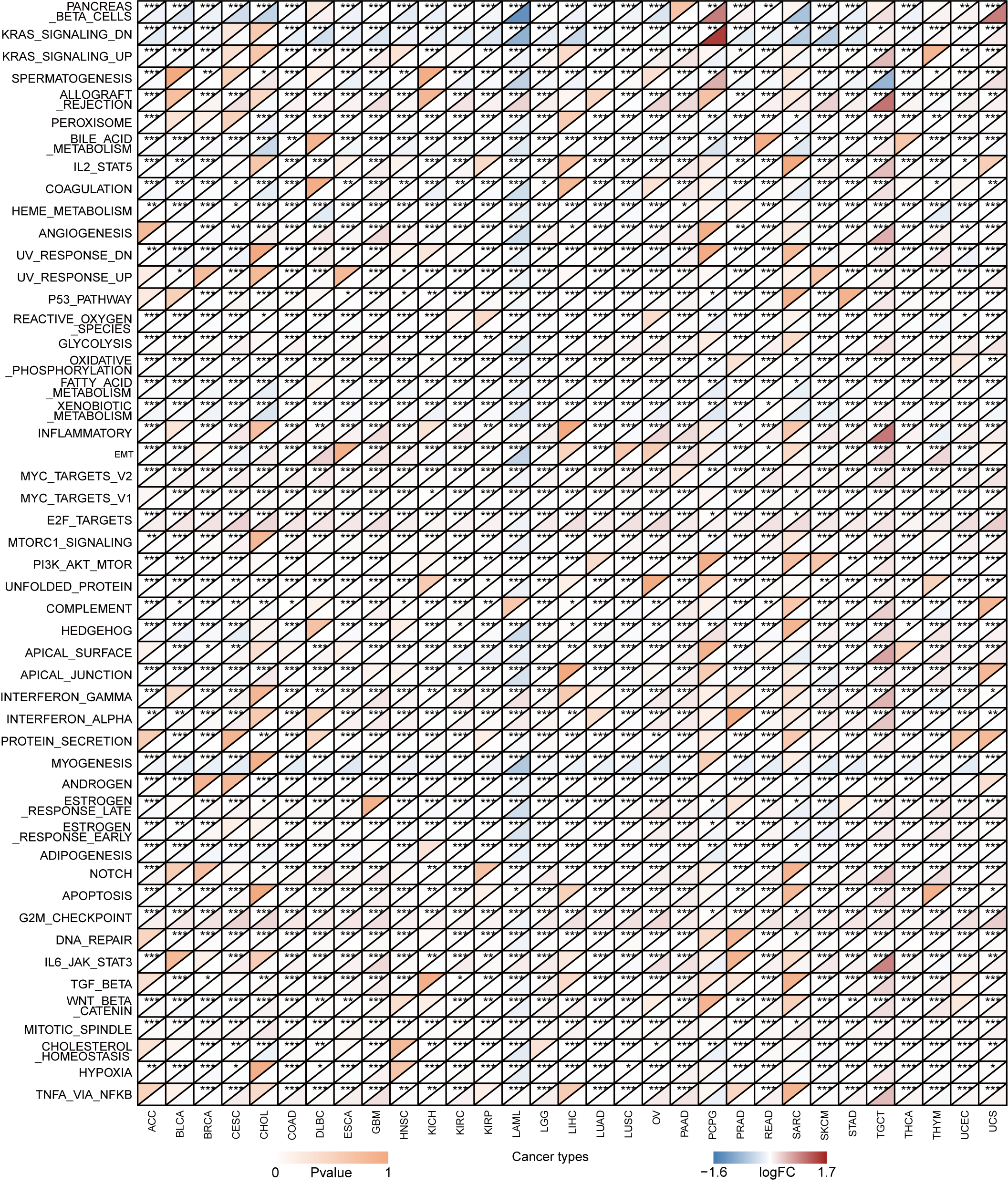

Supplement: Supplementary file 4 — (TIF 6297 KB) [file 12672_2024_1093_MOESM4_ESM.tif]

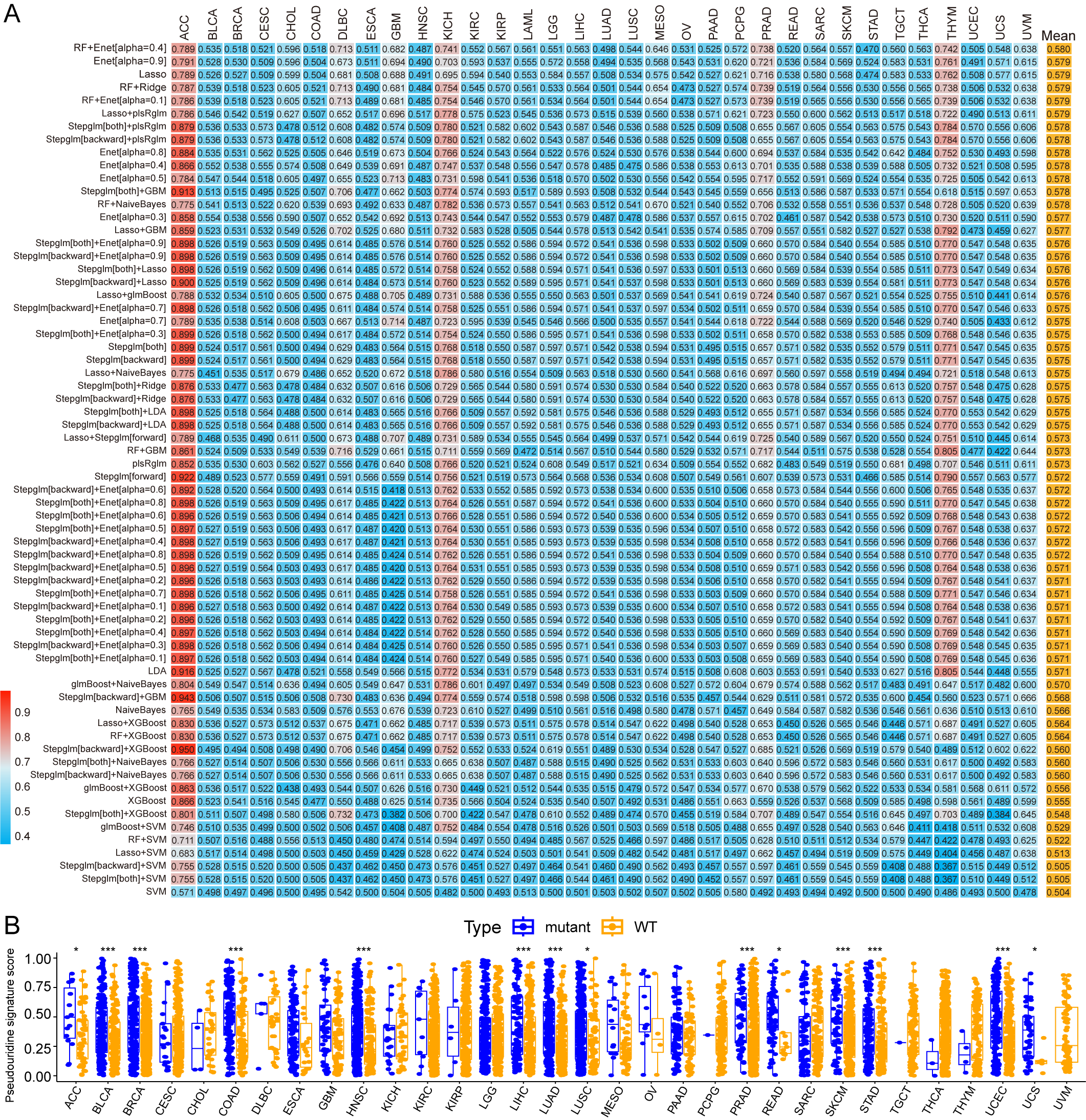

Supplement: Supplementary file 5 — (TIF 8448 KB) [file 12672_2024_1093_MOESM5_ESM.tif]

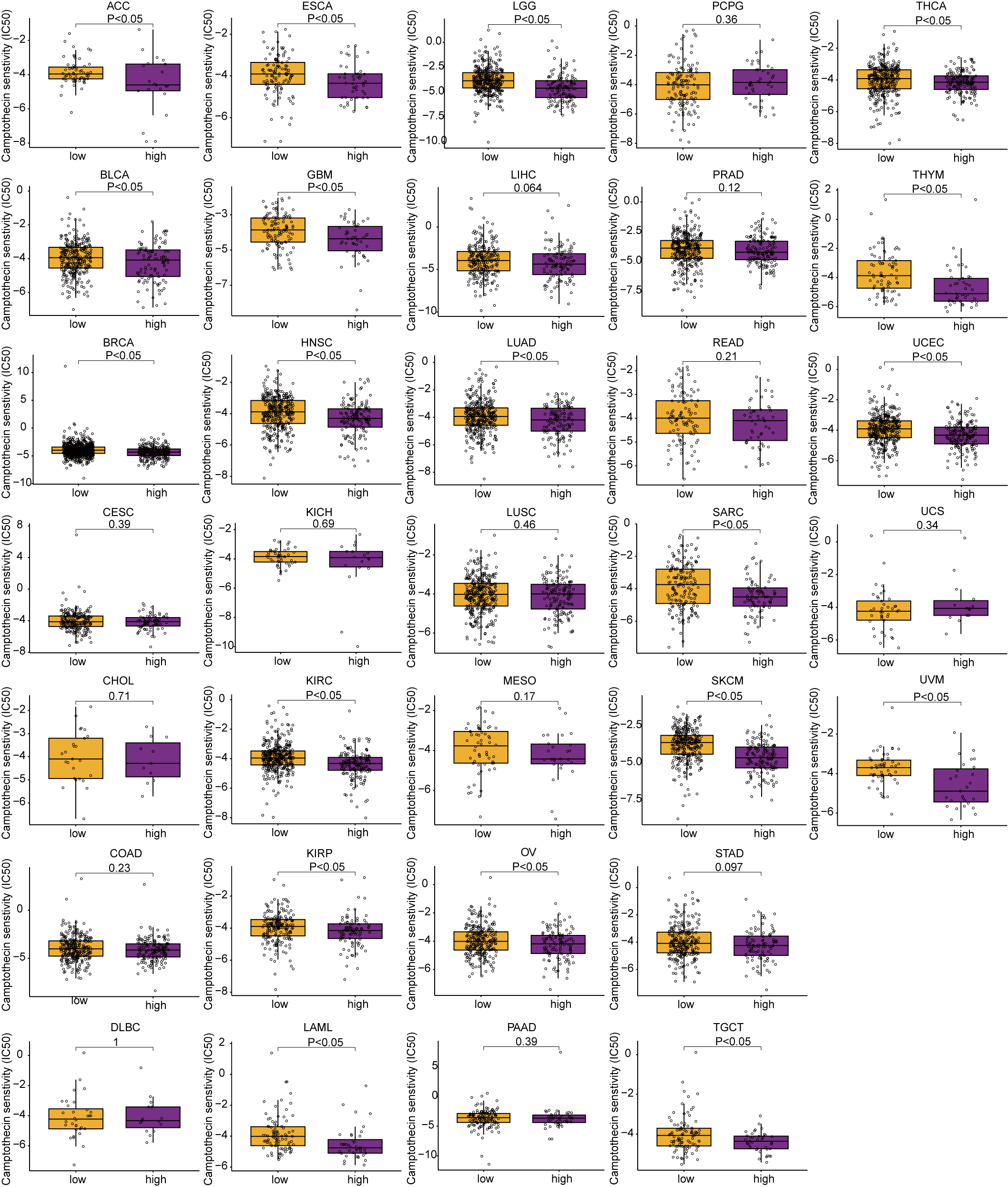

Supplement: Supplementary file 6 — (TIF 2710 KB) [file 12672_2024_1093_MOESM6_ESM.tif]

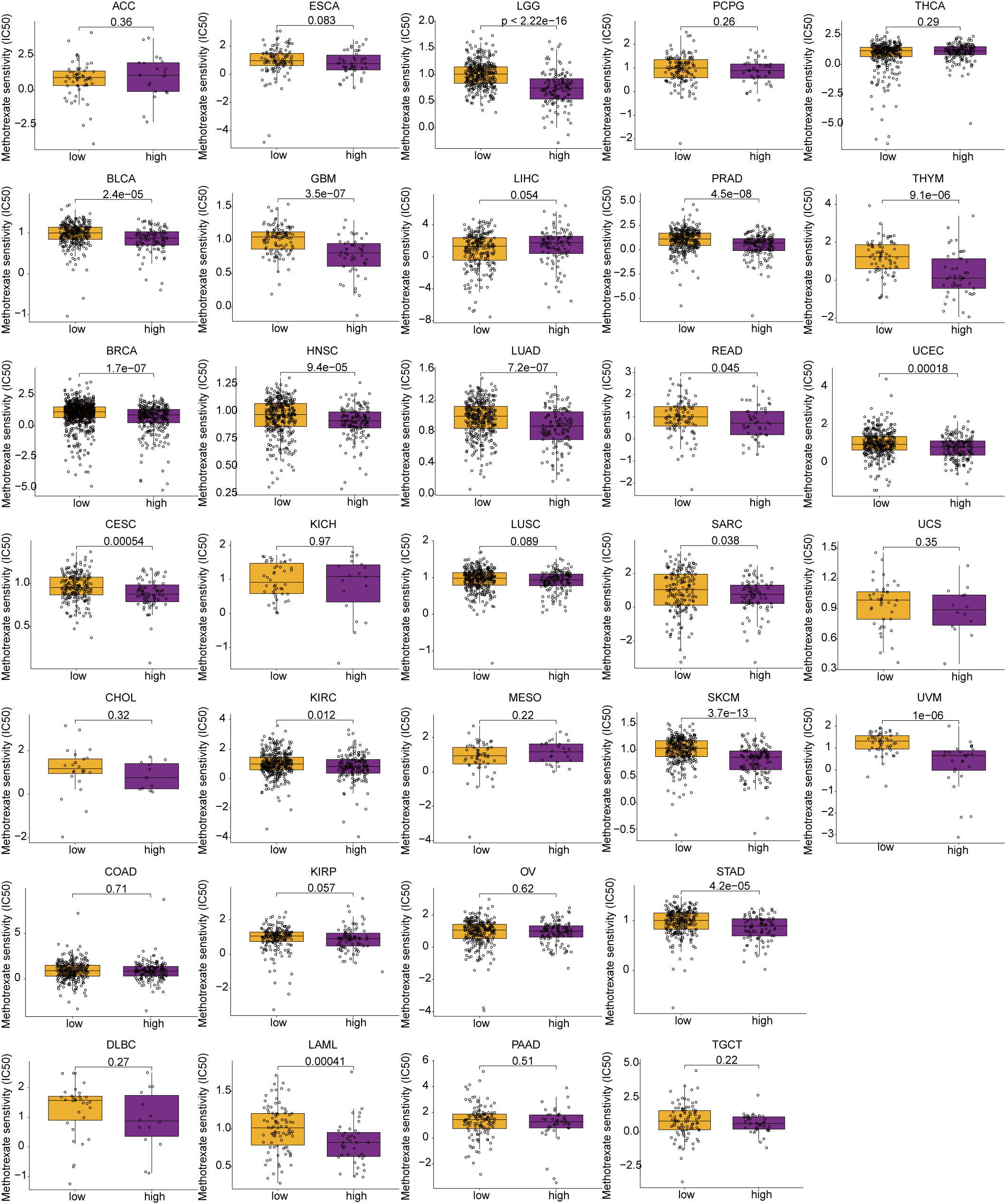

Supplement: Supplementary file 7 — (TIF 2682 KB) [file 12672_2024_1093_MOESM7_ESM.tif]

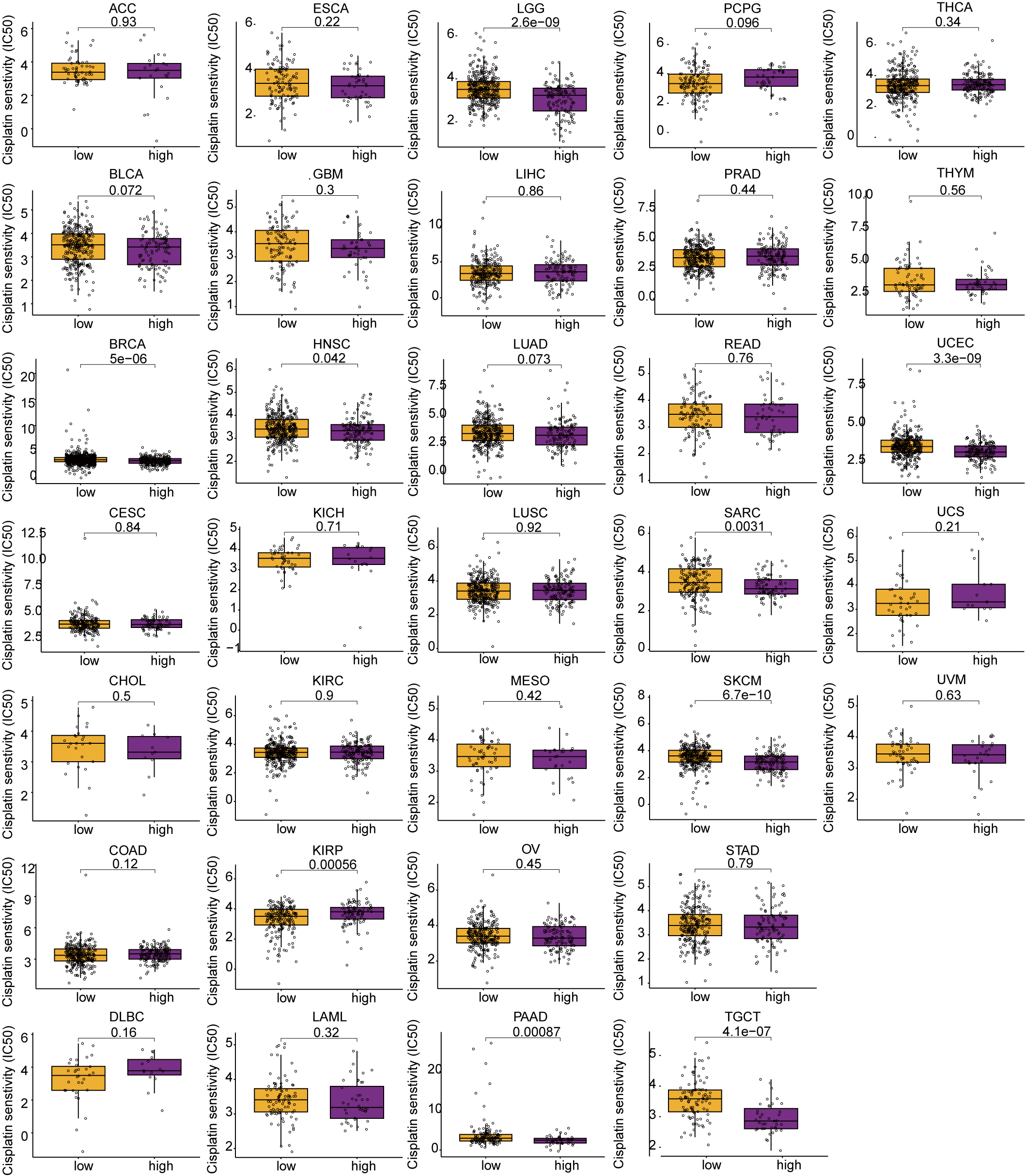

Supplement: Supplementary file 8 — (TIF 2682 KB) [file 12672_2024_1093_MOESM8_ESM.tif]

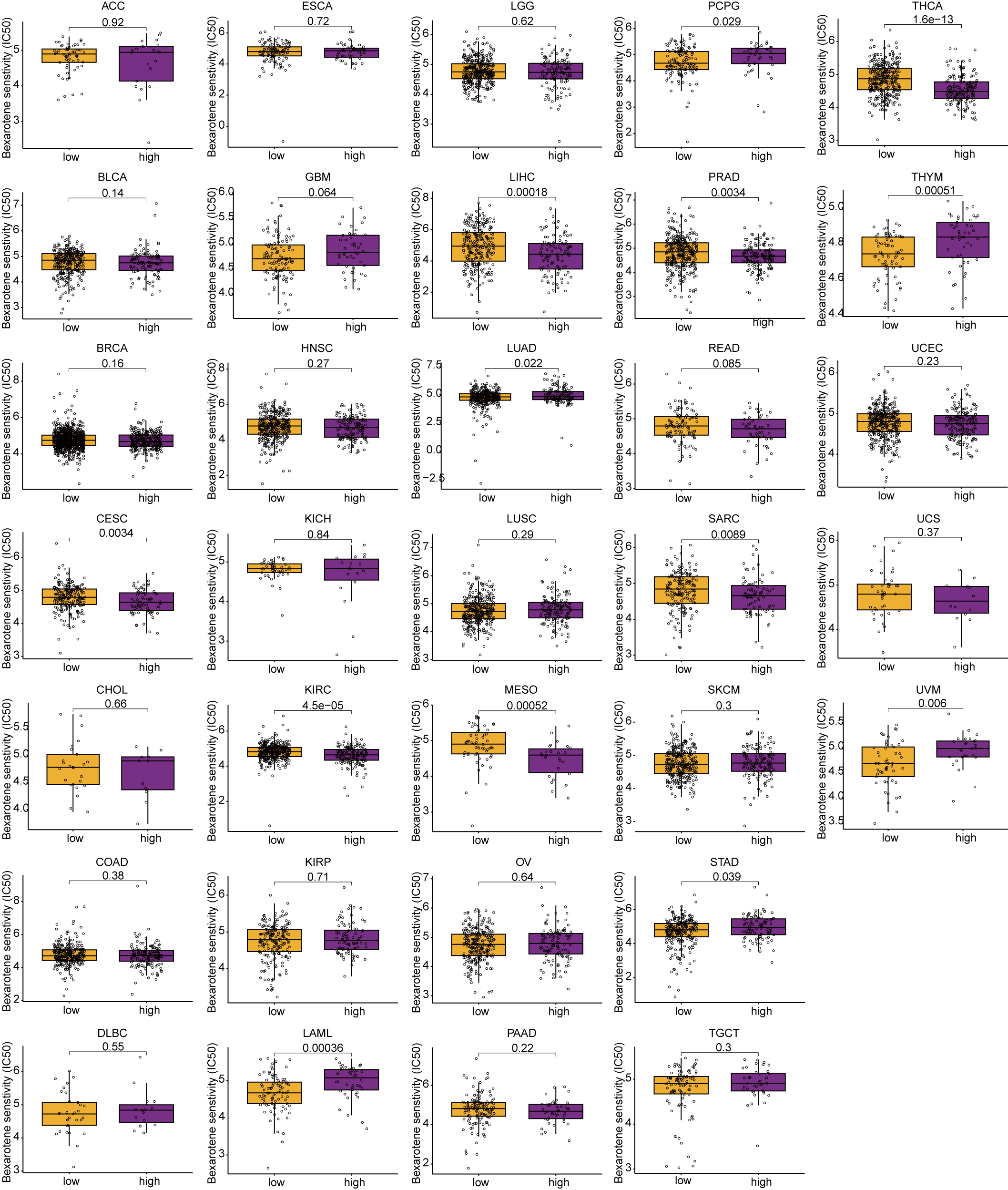

Supplement: Supplementary file 9 — (TIF 2682 KB) [file 12672_2024_1093_MOESM9_ESM.tif]
