# Supplementary material for: Multiomics and machine learning-based analysis of pancancer pseudouridine modifications
Source: Discov Oncol. 2024 Aug 20;15:361. doi: 10.1007/s12672-024-01093-y (PMC11335713; doi:10.1007/s12672-024-01093-y)
Supplement: Supplementary file 10 — (DOCX 21 KB) [file 12672_2024_1093_MOESM10_ESM.docx]

| cancer type | | normal | | tumor | Total |
| --- | --- | --- | --- | --- | --- |
|  |  | GTEx | TCGA | TCGA | |
| ACC | Adrenocortical Cancer | 128 | 0 | 79 | 207 |
| BLCA | Bladder Cancer | 9 | 19 | 411 | 439 |
| BRCA | Breast Cancer | 179 | 113 | 1104 | 1396 |
| CESC | Cervical Cancer | 10 | 3 | 306 | 319 |
| CHOL | Bile Duct Cancer | 0 | 9 | 36 | 45 |
| COAD | Colon Cancer | 308 | 41 | 471 | 820 |
| DLBC | Large B-cell lymphoma | 444 | 0 | 48 | 492 |
| ESCA | Esophageal Cancer | 653 | 11 | 162 | 826 |
| GBM | Glioblastoma | 1152 | 5 | 168 | 1325 |
| HNSC | Head and Neck Cancer | 0 | 44 | 502 | 546 |
| KICH | Kidney chromophobe | 28 | 24 | 65 | 117 |
| KIRC | Kidney Clear Cell Carcinoma | 28 | 72 | 535 | 635 |
| KIRP | Kidney Papillary Cell Carcinoma | 28 | 32 | 239 | 349 |
| LAML | acute myeloid leukemia | 70 | 0 | 151 | 221 |
| LGG | Lower Grade Glioma | 1152 | 0 | 529 | 1681 |
| LIHC | Liver Cancer | 110 | 50 | 374 | 534 |
| LUAD | Lung adenocarcinoma | 288 | 59 | 526 | 873 |
| LUSC | Lung squamous cell carcinoma | 288 | 49 | 501 | 838 |
| MESO | Mesothelioma | 0 | 0 | 86 | 86 |
| OV | Ovarian Cancer | 88 | 0 | 379 | 467 |
| PAAD | Pancreatic Cancer | 167 | 4 | 178 | 349 |
| PCPG | Pheochromocytoma & Paraganglioma | 0 | 3 | 183 | 186 |
| PRAD | Prostate Cancer | 100 | 52 | 499 | 651 |
| READ | Rectal Cancer | 308 | 10 | 167 | 485 |
| SARC | Sarcoma | 2 | 0 | 263 | 265 |
| SKCM | Melanoma | 812 | 1 | 471 | 1284 |
| STAD | Stomach Cancer | 174 | 32 | 375 | 581 |
| TGCT | Testicular Cancer | 165 | 0 | 156 | 321 |
| THCA | Thyroid Cancer | 279 | 58 | 510 | 847 |
| THYM | Thymoma | 444 | 2 | 119 | 565 |
| UCEC | Endometrioid Cancer | 78 | 35 | 548 | 661 |
| UCS | Uterine carcinosarcoma | 78 | 0 | 56 | 134 |
| UVM | Ocular melanoma | 0 | 0 | 80 | 80 |
| Total |  | 7570 | 728 | 10277 | 18625 |

Supplementary Table 1: The number of 33 tumour samples and corresponding GTEx control samples explored in this study.
