# Supplementary material for: Multiomics and machine learning-based analysis of pancancer pseudouridine modifications
Source: Discov Oncol. 2024 Aug 20;15:361. doi: 10.1007/s12672-024-01093-y (PMC11335713; doi:10.1007/s12672-024-01093-y)
Supplement: Supplementary file 11 — (DOCX 20 KB) [file 12672_2024_1093_MOESM11_ESM.docx]

**Supplementary Material**

**Supplementary Table 1**: The number of 33 tumour samples and corresponding GTEx control samples explored in this study.

**Supplementary Figure 1: Survival difference analysis. A-G** K‒M survival analysis of pseudouridine-related pathways in LUAD, BRCA, LGG, LUSC, PAAD, and SARC based on OS.

**Supplementary Figure 2: Differences in the expression patterns of PUSs in tumor and paracancerous tissues.** Blue represents the gene expression values in the positive difference group, and orange represents the expression values in the tumor tissues. **A-F** are denoted as PUS3, PUS1, PUS10, RPUSD4, TRUB1, and TRUB2, respectively.

**Supplementary Figure 3: Genomic aberrations in PUSs.** **A** Each pie chart represents the CNV composition of each PUS in one tumor. Different colors represent the percentage of different types of mutations. Hete amp. and Hete dele. denote samples with copy number heterozygous amplification or deletion. Homo amp and Homo dele denote samples with copy number homozygous amplification or deletion. **B** Waterfalls showing mutations in UCEC for 8 PUSs. **C** Based on the TCGA dataset, the PUSs methylation differences in tumor and paracancerous tissues. Colors represent values for tumors over paracancerous tissues. Circles represent FDR values. **D** Bubble plots demonstrating the correlation between PUSs expression and immune cell infiltration in KIRC. The threshold was FDR <= 0.05. Colors indicate the magnitude of positive and negative correlations.

**Supplementary Figure 4:** **Characterization of hallmark gene sets for tumor and nontumor tissues (including TCGA and GTEx data) using the ssGSEA algorithm.** Triangles above and below in each heatmap square indicate the significance and degree of difference (tumor vs nontumor), respectively. Thresholds are p values < 0.05 (*p < 0.05; ** p < 0.01; *** p < 0.001)

**Supplementary Figure 5: Construct pseudouridine signature model and TP53 state. A** Heatmap demonstrating the prognostic C-index in tumors based on machine learning algorithms to construct pseudouridine signature model (the remaining 72 results). **B** Differences in pseudouridine signature scores between the TP53 mutant and wild-type groups.

**Supplementary Figure 6:** Intergroup differences in IC50 values of the chemotherapeutic agent camptothecin predicted by the ‘pRRophetic’ method in different tumors.

**Supplementary Figure 7:** Intergroup differences in IC50 values of the chemotherapeutic agent methotrexate predicted by the ‘pRRophetic’ method in different tumors.

**Supplementary Figure 8:** Intergroup differences in IC50 values of the observed chemotherapeutic agent cisplatin predicted by the ‘pRRophetic’ method in different tumors.

**Supplementary Figure 9:** Intergroup differences in IC50 values of the observed chemotherapeutic agent bexarotene predicted by the ‘pRRophetic’ method in different tumors.
