# Supplementary material for: Multiomics and machine learning-based analysis of pancancer pseudouridine modifications
Source: Discov Oncol. 2024 Aug 20;15:361. doi: 10.1007/s12672-024-01093-y (PMC11335713; doi:10.1007/s12672-024-01093-y)
Supplement: Supplementary file 12 — (DOCX 21 KB) [file 12672_2024_1093_MOESM12_ESM.docx]

Introduction to 12 Machine Learning Algorithms

1. Lasso

Least absolute shrinkage and selection operator (Lasso) regression, a common linear regression method, employs L1 regularization in conjunction with ordinary least squares to eliminate the coefficients of uncorrelated or redundant features. This process enables Lasso regression to automatically identify the most significant features that are correlated with the target variable. One of the key advantages of Lasso regression is its ability to overcome the limitations of local optimal estimation in least squares estimation and stepwise regression.

1. Ridge

Ridge regression is a method for addressing linear regression issues, particularly when multicollinearity exists between predictor variables. The fundamental premise of Ridge regression is a modified least squares estimation approach, which constrains the model parameters by incorporating an L2 regularisation term into the loss function. The advantages of Ridge regression include the ability to reduce overfitting and to effectively deal with the problem of multicollinearity between predictor variables, thereby making the model parameter estimates more stable.

1. Enet Regression

Elastic net (Enet) regression combines the advantages of L1-regularised LASSO and L2-regularised ridge regression, aiming at selecting important features while reducing noise in the data. The advantage of the algorithm is that it can effectively handle datasets with multicollinearity while performing feature selection. The disadvantage, on the other hand, is that parameter tuning can be complex and cross-validation is required to determine the optimal parameters.

1. SVM

The fundamental concept of support vector machine (SVM) is to map the data in a high-dimensional space to a low-dimensional space, and to divide the data points into two classes by identifying an optimal solution. This optimal solution must ensure that the two classes of data are completely separated, and that the partition gap between the two classes of data is clearly visible in space. Furthermore, in order to address nonlinear problems, SVM introduces the concept of soft spacing, which entails identifying the optimal value while allowing for a certain degree of error. As a robust classification algorithm, SVM necessitates the pre-processing of the input matrix, encompassing feature extraction, data normalisation, and other techniques, to enhance the performance of SVM.

1. glmBoost

The gradient boosted generalised linear model (glmBoost) is a hybrid model that combines the advantages of gradient boosting with the flexibility of a generalised linear model. glmBoost is constructed by iteratively adding weak models, each of which is constructed in the direction of reducing the residuals of the previous model. These weak models are usually simple models such as small decision trees or linear regression models. However, when these weak models are combined in a specific way, they can form a powerful predictive model. The training process of glmBoost involves a process known as functional gradient descent, in which the model is optimised along the direction of the negative gradient of the loss function in the function space. At each iteration, the model attempts to identify a novel weak model that optimally aligns with the negative gradient of the current model's residuals, until a predefined number of iterations is reached or the model performance is optimal. glmBoost exhibits a significant advantage in its capacity to address intricate nonlinear relationships. Furthermore, the algorithm is adept at handling heterogeneous data types, including the management of missing values and outliers.

1. LDA

Linear discriminant analysis (LDA) is a classical supervised learning algorithm that is primarily employed for classification and dimensionality reduction. The objective of LDA is to project data into a novel space, where data belonging to distinct classes are separated as extensively as feasible, while data belonging to the same class are clustered as closely as possible, with the intention of enhancing the accuracy of the classification. The mathematical principles of LDA are relatively simple, easy to understand and implement, computationally efficient, and the classification results are comparable to those of more complex models. However, LDA assumes that the data are normally distributed and also assumes that the different categories of data have the same covariance matrix, which may not always be the case. LDA may not be directly applicable when the number of features is larger than the number of samples. Despite these limitations, LDA remains a valuable tool, particularly in the preprocessing stage, for dimensionality reduction to facilitate subsequent classification algorithms. In practice, LDA is often employed in conjunction with other machine learning algorithms to enhance the overall prediction performance.

1. plsRglm

The combination of the features of Partial Least Squares Regression (PLS) and Generalised Linear Models (GLM) in plsRglm (Partial Least Squares Regression with Generalised Linear Models) is particularly suited to datasets with a large number of predictor variables. This approach is particularly suitable for the presence of multicollinearity in a dataset, where there is a high degree of correlation between multiple predictor variables. In traditional regression analysis, the presence of multicollinearity can result in unstable model estimates, which in turn impairs the model's predictive capacity. In contrast, the PLS method employed by plsRglm facilitates dimensionality reduction, thereby capturing the majority of the information present in the original variables. The inputs to the GLM are then used to build the predictive model. plsRglm is capable of handling not only continuous response variables, but also binary, count, and other types of response variables. This is achieved by using different link functions and distributional assumptions. In conclusion, plsRglm is a highly effective tool that combines the dimensionality reduction capabilities of PLS with the flexibility of GLM, providing an optimal approach to building predictive models, particularly when dealing with high-dimensional data and in the presence of multicollinearity issues.

1. RF

The random forest (RF) algorithm improves the accuracy and stability of a model by constructing multiple decision trees and integrating their predictions. The basic principle of this approach is twofold: bootstrap sampling and random feature selection. During the sampling process, the RF algorithm constructs each decision tree by iteratively taking samples from the original dataset and allowing repetition. In addition the algorithm allows each decision tree to be trained on a different subset of the data, thus enhancing model diversity. This further increases the variability between models and helps to reduce the risk of overfitting. The predictions of the RF are derived by aggregating the predictions of all decision trees. In classification problems, this is usually achieved through a majority voting mechanism. In regression problems, the average of the predictions of all decision trees is computed. RF is a robust algorithm that can handle large amounts of data and features, and is insensitive to outliers and noise. It provides highly accurate predictions even in the face of multiple composite models. In addition, RF evaluates the relative importance of each feature in the prediction. In conclusion, Random Forest is popular for various machine learning tasks due to its excellent performance and ease of use.

1. GBM

The basic concept of the gradient boosting machine (GBM) algorithm is to combine multiple weak models (usually decision trees) and improve them incrementally through an iterative approach, resulting in a model with strong predictive power. In GBM, each iteration produces a new weak predictive model that focuses on data points that the previous model failed to predict accurately. The strength of GBM lies in its flexibility and robust performance. It can handle a variety of data types, including classification and regression problems. However, the GBM model training process can be relatively time-consuming because it requires building multiple models incrementally. Secondly, GBM models may be overfitted when the amount of data is limited or the data quality is poor. Therefore, proper parameter tuning must be implemented to build effective GBM models. Despite these challenges, GBM has been highly successful in practice, providing efficient implementations and incorporating a number of regularisation techniques to prevent overfitting and improve model performance.

1. XGBoost

eXtreme Gradient Boosting (XGBoost) enhances efficiency and accuracy by optimising the computational process of distributed gradient boosting. At the system level, it performs optimisations, including support for parallel processing and cache optimisations, which allow the algorithm to process large-scale data in a timely manner. Additionally, XGBoost introduces second-order derivative approximation to identify the minimum of the loss function with greater accuracy, and supports custom loss functions. It is noteworthy that XGBoost incorporates cross-validation and regularisation techniques to enhance the model's generalisation capabilities. In practice, in addition to its various types of prediction capabilities (including regression and classification), XGBoost is also suitable for feature importance scoring.

1. NB

Naive Bayes (NB) is a straightforward probabilistic classification algorithm based on Bayes' theorem. The fundamental assumption of the algorithm is that each feature is independent of other features within the same category. This implies that, given a category, the probability of a feature's occurrence can be calculated, which can then be employed for subsequent instance prediction. Although the independence assumption of the NB algorithm is often not borne out in the real world, this algorithm remains highly effective in practice. This is because even if the assumption of independence between features is not entirely accurate, the probabilistic model can still capture sufficient information to make accurate predictions. An important advantage of the NB algorithm is that it is computationally highly efficient. Due to the independence between features, the algorithm is able to learn and make predictions in a relatively short period of time. Notably, the NB algorithm is insensitive to missing data and is able to handle a large number of features, which is especially useful when dealing with high-dimensional data. In conclusion, the NB algorithm is a powerful and flexible tool that has been widely used in the field of machine learning.

1. stepGLM

The stepGLM algorithm is a stepwise regression method for constructing generalised linear model. The stepGLM algorithm is capable of constructing simplified models by automatically selecting important predictor variables. This is achieved through an iterative process of evaluation and selection, whereby variables are added or removed from the model at each step. This approach, which involves a stepwise procedure, is more efficient than a fully fitted model, particularly when the number of variables is large. Furthermore, the method is applicable to variables with multiple distributions, including binomial and Poisson distributions. Nevertheless, the selection of an excessive number of variables may result in the generation of an overly complex model. Furthermore, the final model may be contingent upon the initial selection of variables. A potential consequence of a biased variable selection process is an inaccurate prediction. Nevertheless, the stepGLM algorithm is an effective variable selection mechanism when dealing with high-dimensional data. In practical applications, it can be combined with cross-validation methods to assess the predictive ability of the model.
